# Strong selective environments determine evolutionary outcome in time‐dependent fitness seascapes

**DOI:** 10.1002/evl3.284

**Published:** 2022-05-26

**Authors:** Johannes Cairns, Florian Borse, Tommi Mononen, Teppo Hiltunen, Ville Mustonen

**Affiliations:** ^1^ Organismal and Evolutionary Biology Research Programme (OEB), Department of Computer Science University of Helsinki Helsinki 00014 Finland; ^2^ Department of Microbiology University of Helsinki Helsinki 00014 Finland; ^3^ Department of Biology University of Turku Turku 20014 Finland; ^4^ Institute of Biotechnology University of Helsinki Helsinki 00014 Finland

**Keywords:** Antibiotic resistance, *Escherichia coli*, experimental evolution, fitness seascape, fluctuating selection, microbial evolution, phage resistance, pleiotropy, sub‐MIC, time‐dependent selection

## Abstract

The impact of fitness landscape features on evolutionary outcomes has attracted considerable interest in recent decades. However, evolution often occurs under time‐dependent selection in so‐called fitness seascapes where the landscape is under flux. Fitness seascapes are an inherent feature of natural environments, where the landscape changes owing both to the intrinsic fitness consequences of previous adaptations and extrinsic changes in selected traits caused by new environments. The complexity of such seascapes may curb the predictability of evolution. However, empirical efforts to test this question using a comprehensive set of regimes are lacking. Here, we employed an in vitro microbial model system to investigate differences in evolutionary outcomes between time‐invariant and time‐dependent environments, including all possible temporal permutations, with three subinhibitory antimicrobials and a viral parasite (phage) as selective agents. Expectedly, time‐invariant environments caused stronger directional selection for resistances compared to time‐dependent environments. Intriguingly, however, multidrug resistance outcomes in both cases were largely driven by two strong selective agents (rifampicin and phage) out of four agents in total. These agents either caused cross‐resistance or obscured the phenotypic effect of other resistance mutations, modulating the evolutionary outcome overall in time‐invariant environments and as a function of exposure epoch in time‐dependent environments. This suggests that identifying strong selective agents and their pleiotropic effects is critical for predicting evolution in fitness seascapes, with ramifications for evolutionarily informed strategies to mitigate drug resistance evolution.

**Impact Summary**

Evolution is often inspected as positive or negative selection for a heritable trait improving or decreasing fitness in a particular environment, causing the frequency of the trait to increase or decrease in the population of an organism. However, there is considerable complexity to this process. Notably, the heritable trait increasing fitness in a particular environment may affect fitness in other environments. This is important since in real‐life scenarios selective environments tend to change over time. Are certain temporal sequences of environments particularly beneficial or detrimental to an organism harboring specific heritable traits? This question is not merely of theoretical interest. For instance, climate change involves changing environmental conditions, and it is important to understand what types of traits may become enriched in populations to predict the response of different organisms to climate change. Moreover, in the treatment of cancers and pathogens, temporal sequences of drugs could be exploited to steer evolution of the target cell population in a desired direction, such as elimination of a pathogen and prevention of the emergence of drug resistance. Here, we used a microbial model system to investigate in vitro the factors driving the evolutionary response across changing environments. We used antibiotics and a virus to model different selective environments, combining the antibiotics into different temporal sequences. We found that only a couple of the environments drove the evolutionary response to all the environmental conditions applied. They caused stronger selection not only for specific adaptations targeted at them but also altered phenotypic and genetic properties relevant to withstanding the other environments. Their time of exposure in the temporal sequence also affected the level of resistance to them and the other agents. These results indicate that identifying driver agents and their phenotypic effects is critical to assessing the evolutionary response of populations exposed to changing environments.

Despite the high dimensionality of fitness landscapes (McCandlish 2011), the genomes of evolving organisms are frequently visualized in fitness landscapes with low‐fitness valleys and high‐fitness peaks (Weinreich et al. 2006; de Visser et al. 2018). The initial position on the landscape depends on the genomic background, which varies between lineages within a population, and determines the mutations with positive fitness consequences. Once a mutation occurs in a phenotypic or genotypic direction in the fitness landscape that makes it beneficial, it will be selected, causing movement upward from a valley or toward a peak. This position, in turn, determines the subsequent mutations with high selection coefficients. The population can eventually become trapped on a local peak or reach a global peak depending on the starting position, traversed path, and ruggedness of the landscape. The concept of a three‐dimensional fitness landscape is based on the realization that the fitness effects of mutations virtually always depend on the genomic background (pervasive epistasis). Typically, the topology of the landscape is presented as static. This may hold in a minimal setup with the evolution of a single gene in a stable environment and when complications such as frequency‐dependent selection are not present. What features of such static landscapes affect the predictability of evolution has become an active research field (de Visser and Krug 2014; Lässig et al. 2017). However, in many realistic scenarios, the targets of selection change over time, causing the fitness landscape also to change over time, becoming a fitness seascape (Mustonen and Lässig 2009; Lässig et al. 2017). Therefore selection itself should be regarded as a time‐dependent force in evolutionary dynamics as discussed in the classical population genetic theory by Wright, Kimura, Ohta, Gillespie, and others (Wright 1948; Kimura 1954; Gillespie 1972; Ohta 1972; Gillespie 1992). For instance, mutations improving adaptation of a trait under directional selection often have deleterious consequences on traits under stabilizing selection, an example of pleiotropy, causing selection to alternate between mutations improving the adaptive trait and mutations compensating for the cost on the conserved trait. Moreover, the concept of fitness seascape inherently captures evolution along time‐dependent selective environments. This is sometimes called fluctuating selection (Bell 2010; Dean et al. 2017), which also refers to the specific situation where the strength of selection by particular selective pressures displays a pattern of recurrence over time, such as in negative frequency‐dependent selection (Hall et al. 2011), rather than different selective environments occurring one after each other.

In fitness seascapes, rather than the stepwise refinement of a single trait depending on a limited set of epistatic interactions, the mutational path is better characterized as a serpentine path where previous adaptations can have varied effects on where the genome lands in each time‐dependent landscape. One consequence of this is that directional selection improving a particular trait can be stronger for time‐invariant compared to time‐dependent environments, although this is modulated by the frequency and duration of exposure to any given environment. In particular, although infrequent changes in the fitness seascape open the opportunity for adaptive substitutions driven by positive selection, too rapidly changing seascapes hinder the adaptive flux that can be sustained by closing each opportunity before a typical substitution event has completed (Takahata et al. 1975; Mustonen and Lässig 2008). Moreover, mutations improving an adaptive trait in a particular selective environment often influence other traits (i.e., display pleiotropy), which may make the organism either more or less adaptive to a subsequent selective environment compared to the initial state. Pleiotropic effects have been shown to be prevalent in multiple systems, including microbes evolving antimicrobial resistance (Rosenkilde et al. 2019). Strong pleiotropic effects can have dramatic effects on the evolutionary outcome as key traits may improve or deteriorate even in the absence of direct selection (Martin and Lenormand 2015; Harmand et al. 2017).

In time‐dependent environments, epistasis and pleiotropy are expected to give rise to historical contingency of evolution whereby the mutations that are adaptive in the current environment are contingent on the adaptive mutations accrued in the previous environment(s) (Kim et al. 2014; Nichol et al. 2015; Yen and Papin 2017). This should be seen as differences in the mutational paths and profiles as well as in phenotypic outcomes when the order of the environments is altered, with some sequences constraining and others potentiating evolution. From a statistical viewpoint, the variance of outcomes should therefore be greater than expected by chance. A low deviation from the null expectation would indicate a negligible role for epistasis and pleiotropy in adaptation. For instance, in the case of strong selective pressures, selection may be strong enough and sustained for long enough to allow for fixation of the relevant mutations before the environment changes again, causing the mutational profile and adaptive trait outcome in a time‐dependent environment to be simply an aggregate of exposure to each environment in isolation. However, because strong adaptations often have deleterious consequences on conserved traits (among the most frequent modes of pleiotropy), accruing a succession of strong adaptations each accompanied by a fitness cost may prove deleterious after a particular set of environments. This could cause the extinction probability to increase as a function of the number of environments. A dependence of extinction dynamics on the environmental sequence, in turn, could indicate a stronger role for epistasis or pleiotropy, among other factors (i.e., demographic decline or epigenetic changes caused by previous environment).

Even though evolution in real‐world systems typically occurs in dynamic fitness seascapes rather than static landscapes, evolutionary dynamics in fitness seascapes remain relatively poorly understood (Jasmin and Kassen 2007; Boyer et al. 2021). Among the underexplored questions features the relative contribution of directional selection and pleiotropy on the evolutionary outcome in time‐invariant versus time‐dependent environments. Moreover, the extent to which the environmental sequence determines the evolutionary outcome in time‐dependent environments has not been comprehensively examined. The relative role of these processes influences the predictability of evolution and determines the conditions where adaptation is constrained or enhanced, which is key also for any practical efforts to guide evolution, such as in evolutionarily informed strategies to treat cancer or mitigate antimicrobial resistance (Nichol et al. 2015; Roemhild and Schulenburg 2019; Tyers and Wright 2019).

Here, we designed a minimal setup for assessing evolutionary outcomes in fitness seascapes, comparing time‐invariant and time‐dependent environments, and including among the latter all possible permutations of environmental orders. We used a microbial model system exposing *Escherichia coli* at high replication to four environmental epochs consisting of three different antimicrobials at subinhibitory levels and an antimicrobial‐free environment (Fig. 1). We also performed the full experiment in the presence of a bacteriophage to investigate the modulatory impact of an added layer of selective pressure, resulting in a total of 928 bacterial populations. Phages are also of interest by adding another biotic stress to the system as well as representing an alternative type of antibacterial agent, with an increasing trend in real‐life clinical applications of phage therapy. We phenotyped populations over time for adaptation to each selective pressure as well as phenotyping and whole‐genome sequencing clones isolated from populations at the experimental end point. To assess differences in evolutionary outcomes between fitness seascapes, we used a combination of analyses, including information theoretical and machine learning approaches. This enabled us to identify characteristics of fitness seascapes constraining or enhancing adaptation and influencing the predictability of evolution. Our findings have implications for evolutionarily informed strategies to manage populations, such as mitigating antimicrobial resistance evolution.

**Figure 1:**
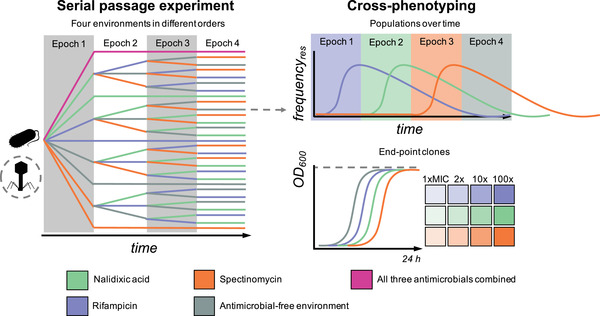
Overview of experimental design to systematically study evolution under fitness seascapes. The main experiment on the left was a 48‐day serial passage experiment where initially isogenic *E. coli* was subjected to a control environment without antimicrobials; time‐invariant environments with single antimicrobials or all three antimicrobials combined; and time‐dependent environments encompassing all permutations of four 12‐day epochs, including the three antimicrobials and one antimicrobial‐free environment. The antimicrobials were used at subinhibitory concentrations (0.5 × MIC). This translates into a fitness cost seen as an intrinsic growth rate (*r*) of the ancestral bacteria relative to the antibiotic‐free environment of 0.88, 0.46, and 0.36 for nalidixic acid, rifampicin, and spectinomycin, respectively. The full experiment was repeated with and without initial introduction of phage representing an alternative type of selection pressure. Each unique treatment combination was replicated 16 times, amounting to a total of 928 independent populations, which were cross‐phenotyped over time against low‐level resistance to each of the antimicrobials. In addition, a single dominant clone was isolated from all surviving end‐point populations (*N* = 900) and phenotyped for growth (optical density [OD] at 600 nm after 24‐hour culture) at several concentrations of each antimicrobial. To investigate the underlying molecular evolution, a subset of 235 clones representing different regimes and divergent low‐level resistance outcomes were also subjected to whole‐genome sequencing.

## Methods

### MODEL ORGANISMS

As a model organism, we used the *E. coli* strain REL606 (Ara^−^) obtained from the Yale Stock Center (*E*. *coli* B ATCC 11303). To enable distinguishing strains from mixed cultures in potential competition assays, we produced an REL607‐like (Ara^+^) mutant by culturing REL606 on minimal arabinose plates (personal correspondence with Richard Lenski). The mutant was verified by Sanger sequencing by a third party (Institute of Biotechnology, University of Helsinki), and has the same point mutation as REL607, allowing the use of arabinose as a carbon source and thereby chromogenic differentiation from REL606 on arabinose agar (Fig. S1). A co‐culture competition assay was used to determine that the two strains did not initially differ in fitness (Fig. S2). Additional details for mutant isolation and the competition assay are described in *Methods* in the Supporting Information and Tables S1‐S3. As virulent bacteriophage, we used T4 strain ATCC 11303‐B4. All culturing steps across experiments were performed at 37°C.

### SERIAL PASSAGE EXPERIMENT

We conducted a 48‐day serial passage experiment consisting of four 12‐day epochs (Fig. 1). To allow an exhaustive exploration of equivalent antimicrobial exposure space, we subjected initially isogenic populations of *E*. *coli* to all permutations of four antimicrobial treatments (one epoch each). This generated a total of 24 unique exposure histories containing the same antimicrobial treatments in different orders. In addition, we included five sequences, three of them featuring the same antimicrobial treatment (i.e., time‐invariant single agent environment) and one containing all antimicrobials combined (i.e., time‐invariant combination environment) across all epochs throughout the experiment, as well as an antimicrobial‐free control environment. The four antimicrobial treatments for the four 12‐day epochs in the permutation (i.e., time‐dependent) environments consisted of three antimicrobial‐containing and one antimicrobial‐free treatment. The antimicrobial‐free treatment was added because antimicrobial‐free periods can have a major effect on resistance dynamics by either reversing prior resistance owing to fitness costs or by potentiating future adaptation through compensatory adaptations or increased genetic heterogeneity. The three antimicrobials were selected based on the previously established susceptibility of *E*. *coli* REL606 and its ability to de novo evolve resistance to them, as well as difference in antimicrobial class, mode of action, and genomic target of resistance mutations. Different antibiotic classes were used to avoid major synergy or antagonism. However, because such effects at weaker levels are extremely common, we considered that they cannot be completely ruled out and did not screen for them at the experimental design phase. The antimicrobials thus selected were nalidixic acid (naphthyridone, quinolone‐like antimicrobial targeting DNA gyrase), rifampicin (rifamycin antimicrobial targeting RNA polymerase), and spectinomycin (aminocyclitol, aminoglycoside‐like antimicrobial targeting 30S subunit of ribosome). In addition to the antimicrobials, the full experiment was performed both with and without the bacteriophage T4, representing an alternative selective pressure or drug type (phage therapy) with a likely even more divergent cellular target and mode of resistance compared to those between the antimicrobial compounds.

The experiment was performed in a deep 96‐well plate setup in the DM1000 medium, which produces approximately 2 × 10^9^ cells mL^−1^ during a 24‐hour culture cycle at 37°C. The experiment was started by adding approximately 10^6^ cells from a 24‐hour culture of *E*. *coli* to a final volume of 500 μL of DM1000 containing the appropriate antimicrobials. The antimicrobials were used at 0.5 × minimum inhibitory concentration (MIC) to cause relatively strong selection for resistance while not immediately killing susceptible cells or causing extinction of the bacterial population in the presence of phage due to synergistic population crash. Details for MIC determination are described in *Methods* in the Supporting Information and Figure S3. This translates into a fitness cost seen as an intrinsic growth rate (*r*) of the ancestral bacteria relative to the antibiotic‐free environment of 0.88, 0.46, and 0.36 for nalidixic acid, rifampicin, and spectinomycin, respectively (Fig. S4). For the phage treatments, 5 × 10^5^ infective particles (constituting a multiplicity‐of‐infection value, or MOI, of 0.5) were subsequently added to the wells. Antibiotic MIC and phage MOI are not comparative measures as virulent phages are multiplying entities that kill susceptible bacteria, whereas antibiotic MIC is a static concentration that kills susceptible bacteria only at concentrations starting from the MIC level. Both phage MOI and antibiotic level relative to MIC affect resistance evolutionary dynamics in important ways. The lower the MOI, the more generations bacteria have to evolve resistance until the majority of the population will encounter the phage. Similar to low MOI levels, sub‐MIC levels of antibiotics that are high enough to cause positive selection for resistance do not require resistance to be immediately present in the standing genetic variation of the population but resistance may evolve over time in a susceptible population. The well plates were cultured at 37°C with constant shaking at 120 r.p.m.

Every 24 hours, 2% (10 μL) of the old culture was transferred to a new well containing fresh medium and the appropriate antimicrobial. However, the phage was allowed to go extinct without replenishment. Every 96 hours, the cultures were freeze‐stored with glycerol at –20°C for later analyses. Details concerning antimicrobial MIC determination using the microdilution method, the culture medium, and antimicrobial concentrations can be found in *Methods* in the Supporting Information. To ensure adequate statistical power, each of the unique treatment combinations (*N* = 58, including 24 antimicrobial sequences and five monotherapy, combination therapy, or control sequences in absence/presence of T4 phage) was repeated 16 times, eight times each for the REL606 and REL607‐like strain. This resulted in a total of 928 unique serially passaged *E*. *coli* populations.

### MEASURING ANTIMICROBIAL AND PHAGE RESISTANCE PHENOTYPES

We quantified the evolution of antimicrobial and phage resistance over time using a pin replicator on agar plate method. In addition, we isolated one individual clone from each experimental end‐point population and phenotyped the clone in experimental conditions for resistance to a range of multiplicities of the MIC value of the ancestral *E*. *coli* strain (1, 2, 10, or 100 × MIC), as well as to the phage. The protocols used are described in detail in *Methods* in the Supporting Information and Figures S5‐S7.

### DNA EXTRACTION, SEQUENCING, AND PRE‐PROCESSING SEQUENCE DATA

DNA extraction for clones from the experimental end point was performed using the Qiaqen DNeasy 96 Blood & Tissue kit according to a custom protocol (detailed protocol below). DNA concentration was measured with the Qubit^®^ 3.0 fluorometer (Thermo Fisher Scientific, Waltham, MA, United States) using the HS assay kit. Whole genome sequencing was performed with the Illumina NovaSeq SP 300 (2 × 150 bp) platform by a third party (Institute for Molecular Medicine Finland, FIMM) according to in‐house protocols. FASTQ files obtained from FIMM were quality controlled with Cutadapt version 1.10 (Martin 2011), including removal of sequencing adapters (with minimum overlap, ‐O, of 10 bp set for adapter match) and trimming sequences by allowing minimum Phred‐scaled quality cutoff (‐q) of 28 for the 30 end of each read, and minimum length of 30 bp. Before and following quality control, the quality of the sequence data was assessed with FastQC version 0.11.8 (http://www.bioinformatics.babraham.ac.uk/projects/fastqc) and MultiQC version 1.7 (Ewels et al. 2016).

### GENOME ALIGNMENT, VARIANT CALLING, AND ANNOTATION

The pipeline used for genomic variant calling and annotation is described in detail in *Methods* in the Supporting Information. The main steps were alignment to the reference genome (NCBI Reference Sequence NC 012967, assembly ASM1798v1) with Bowtie 2 version 2.3.4 (Langmead and Salzberg 2012), variant calling using the Genome Analysis Toolkit (GATK) version 3.8 (McKenna et al. 2010) followed by hard‐filtration, and variant annotation using SnpEff version 4.3i (Cingolani et al. 2012).

### REGRESSION AND MACHINE LEARNING ANALYSES

**Figure 2:**
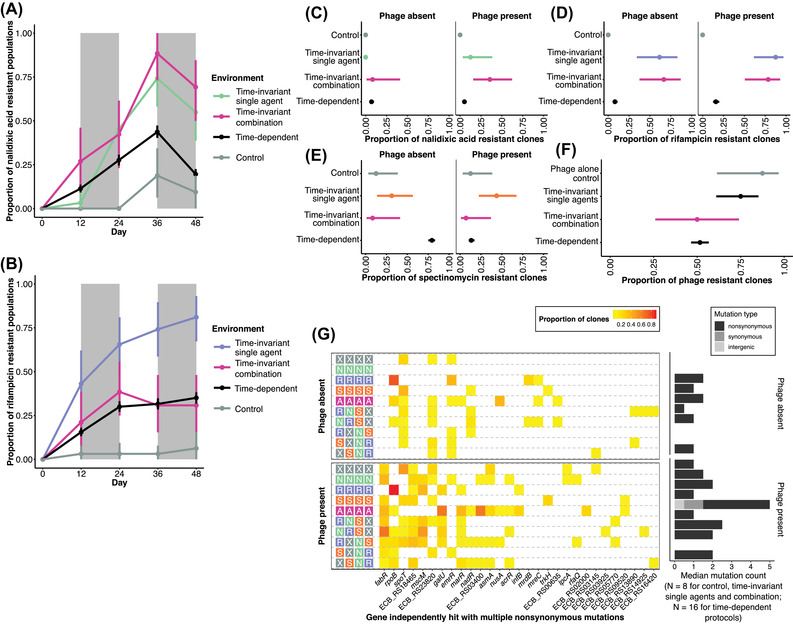
Contingency of phenotypic outcome and mutation landscape on selective regime. Panels A and B show resistance dynamics over time for nalidixic acid and rifampicin, respectively (mean ± bootstrapped 95% confidence intervals; 32 replicates per mean data point; data in presence and absence of phage pooled). Gray rectangles denote epoch boundaries for time‐dependent protocols. Panels C, D, E, and F show low‐level resistance outcomes to nalidixic acid, rifampicin, spectinomycin, and phage T4, respectively, for clones isolated from each population at the experimental end‐point (logistic regression expected value ± 95% confidence intervals; 16 replicates per mean data point). Resistance has been quantified as a binary variable and indicates the ability to grow at levels exceeding the minimum inhibitory concentration of the ancestral bacterial strain. (G) Mutational landscape. The heat map on the left shows the proportion of sequenced clones containing a nonsynonymous (or infrequently synonymous) mutation in a gene recurrently hit in the dataset. The genes have been ordered by total number of hits. The bar plot on the right shows the median mutation count for clones in each history (lack of bar indicates median of 0 mutations). The *y*‐axis labels indicate the antimicrobial therapy protocol with the following encoding for each of the four 12‐day experimental epochs: X = antimicrobial‐free environment; N = nalidixic acid; R = rifampicin; S = spectinomycin; A = all three antimicrobial compounds combined.

**Figure 3:**
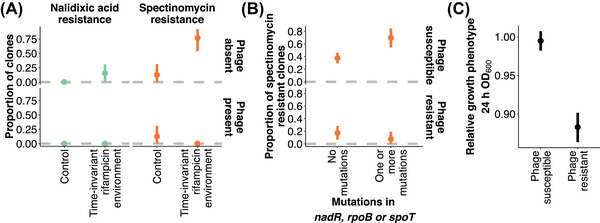
Pleiotropy and fitness effect of resistance. (A) Low‐level nalidixic acid and spectinomycin resistance after 48 days in time‐invariant rifampicin environment (mean ± bootstrapped 95% confidence intervals). (B) Influence of phage resistance on whether mutations in the genes *nadR*, *rpoB*, or *spoT* produce a low‐level spectinomycin resistant phenotype (mean ± bootstrapped 95% confidence intervals). The data are for clones from phage‐exposed environments (for which phage resistance phenotype was determined). (C) Fitness effect of phage resistance (mean ± bootstrapped 95% confidence intervals). Fitness has been quantified as optical density (OD) at 600 nm wavelength after 24‐hour culture in liquid medium. The value has here been related to the mean growth of the clones from the control treatment (absence of antimicrobials and phage). The data for all the figures is for clones isolated from populations at the experimental end point (*N*_total_ = 900), with subset treatments or phenotypes included in a particular analysis indicated in the figure or legend.

**Figure 4:**
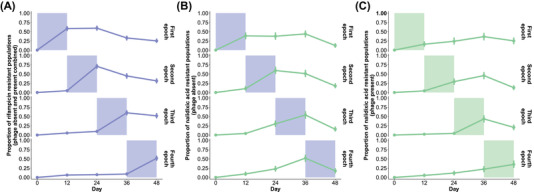
Antimicrobials driving resistance evolution in time‐dependent regimes. (A) Rifampicin resistance over time as a function of rifampicin exposure epoch (both in presence and absence of phage that had no effect on selective antimicrobial as it did for nalidixic acid). (B) Nalidixic acid resistance over time in the absence of phage as a function of rifampicin exposure epoch. (C) Nalidixic acid resistance over time in the presence of phage as a function of nalidixic acid exposure epoch. All the data are shown as mean resistance ± bootstrapped 95% confidence intervals, and are based on *N* = 928 populations. The shaded area indicates the relevant (antimicrobial color code) exposure epoch.

**Figure 5:**
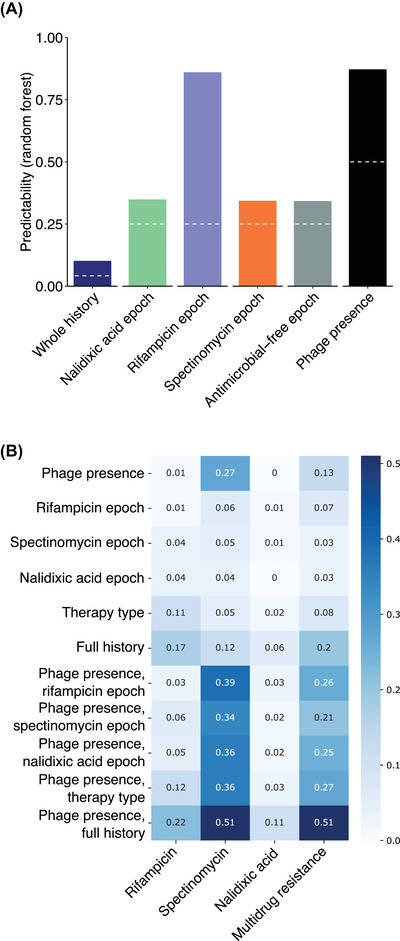
Predictability of past drug exposure and future resistance evolution in time‐dependent regime. (A) Degree of predictive power obtained for antimicrobial and phage exposure past based on phenotypic data from populations and clones at the end‐point of 48‐day serial passage experiment. The white dashed line indicates the level of predictability of the estimated factor by chance. (B) Mutual information between exposure history (rows) and end‐point clone antimicrobial resistance phenotype states (columns). The exposure histories are described at various levels of detail ranging from coarse (phage status and epoch of exposure to an antimicrobial separately) to full information (phage status and antimicrobial epoch order considered simultaneously). For example, the phage presence label of the histories carries substantial information on spectinomycin resistance status of the end points clones.

The regression and machine learning analyses (Figs. 2, 3, 4, and 5A) were performed in the R version 3.6.1 environment (R Core Team 2019). Binomial generalized linear (i.e., logistic regression) models, with binary drug resistance or phage resistance outcome as a response variable and antimicrobial regime (time‐invariant single agent, time‐invariant combination, or time‐dependent protocol), antimicrobial exposure epoch, phage presence, and presence of nonsynonymous mutations in genes of interest as covariates, were performed for experimental end‐point populations or clones using the glm function in base R. Time series resistance data for time‐invariant nalidixic acid and rifampicin environments were analyzed using generalized least squares models, as implemented in the *nlme* package (Pinheiro et al. 2017), assuming AR1 residual correlation structure within replicates. Random forest models for predicting exposure epochs from end‐point phenotypes were generated using the *randomForest* package (Liaw and Wiener 2002). Details about the machine learning procedure are described in *Methods* in the Supporting Information.

### MUTUAL INFORMATION ANALYSES

The analyses were performed in Python, using NumPy and pandas. We first bootstrapped 10,000 individual datasets drawn from the full dataset. Subsequently, the mutual information between experiment outcome and experimental conditions (i.e., environmental history) was calculated on these sets and averaged to a single value.

The following definition of mutual information was used for the calculation:

$$I\left(X;Y\right)=\sum_{y\in Y}\sum_{x\in X}p_{\left(X,Y\right)}\left(x,y\right)\log\left(\frac{p_{\left(X,Y\right)}\left(x,y\right)}{p_{X}\left(x\right)p_{Y}\left(y\right)}\right).$$

The marginal probabilities *p_X_* (*x*) were calculated by counting the number of samples corresponding to every outcome *x*—the level of resistance to the antimicrobial of interest, or the sum of these resistances as in the *multidrug resistance* column in Figure 5B—and dividing the counts by the total number of samples.

The marginal probabilities *p_Y_* (*y*) were calculated by counting the number of samples that were subjected to experimental conditions *y*, and dividing the counts by the total number of samples; these conditions correspond either to exposure to phage; the first epoch a particular antimicrobial has been administered if at all; the type of treatment used, grouping the alternating treatments together while considering the uniform treatments individually; and finally, the full exposure histories. These conditions were then further distinguished additionally by whether the phage had been administered or not. Similarly, the joint probabilities *p_X,Y_* (*x,y*) were calculated by counting the number of samples corresponding to every outcome *x* while subjected to experimental conditions *y*, and dividing the counts by the total number of samples.

## Results

### EVOLUTIONARY DYNAMICS AND OUTCOMES DIFFER BETWEEN AND WITHIN TIME‐INVARIANT AND ‐DEPENDENT ENVIRONMENTS

The evolutionary dynamics and outcomes differed both between and within time‐invariant and time‐dependent environments (Fig. 2A‐F; for statistical outputs for each selective agent at population and clone levels, see Tables S4‐S10). Within the time‐invariant environments, as expected, constant subinhibitory (0.5 × MIC) selection by a single selective pressure (i.e., antimicrobial agent) leads to the emergence of low‐level (1 × MIC) resistance and phage selection to the emergence of phage resistance. Selection was strongest for one antimicrobial agent, rifampicin, with most replicate populations evolving low‐level resistance both in the presence and absence of bacteriophage. The two other agents, nalidixic acid and spectinomycin, depended on the presence of bacteriophage for resistance to evolve to levels detectable for clones from the experimental end point. Even then, low‐level resistance only evolved in a minority of the replicate populations. The development of nalidixic acid and spectinomycin resistance in the presence of phage could be caused by a higher effective MIC (i.e., higher selection coefficient) in the presence of an additional stressor. More sensitive population‐level phenotyping over time, where resistance in a subset of the population could yield a positive signal (while end‐point clones represent dominant population phenotypes), showed stronger nalidixic acid resistance development, with a minor clade in most populations developing low‐level resistance (end‐point phenotyping for one clone from each population could only reveal major clade resistance). This could not be assessed for spectinomycin as the temporal population‐level data were too noisy (high levels of positive signal across environments). This is likely to be the outcome of random genetic variation in minor clades exhibiting low‐level resistance phenotypes.

A time‐invariant environment combining all three antimicrobials showed similar levels of rifampicin resistance and higher levels of nalidixic acid resistance (also in the absence of phage) compared to the single‐antimicrobial environments, whereas spectinomycin resistance failed to evolve. The higher levels of nalidixic acid resistance could either be the outcome of the effective MIC being higher (i.e., higher selection coefficient) in the multidrug (i.e., multistressor) environment compared to the single‐drug environment or pleiotropic effects from rifampicin resistance (inspected further below). Because spectinomycin exhibited the weakest selection pressure out of the three antimicrobials, the failure of spectinomycin resistance to evolve in the multidrug environment may be the outcome of the fitness cost of more readily evolved rifampicin or nalidixic acid resistance further decreasing the selection coefficient for spectinomycin resistance.

Evolution was strongly constrained for two out of the three antimicrobials, rifampicin and nalidixic acid, in the time‐dependent compared to time‐invariable environments, with no to low proportions of replicate populations evolving low‐level resistance. Spectinomycin was a striking exception, with no sign of resistance evolution in the presence of phage but high prevalence of resistance in the absence of phage (see next section for interpretation).

Phenotypic resistances to each of the antimicrobials as well as the phage were associated with recurrent mutations in particular genes (Fig. 2G). Some of these genes have previously been associated with phenotypic resistance to these agents, whereas others have not and are implicated with low‐level phenotypic resistance in this study (for detailed information on these genes and references to previous research, see *Results* in the Supporting Information). There were little signs of differences in recurrent genes between time‐invariant and time‐dependent environments, although the low prevalence of resistance evolution in the latter prevents a thorough statistical testing of this question.

### PLEIOTROPIC AND FITNESS EFFECTS OF STRONG SELECTIVE AGENTS, RIFAMPICIN AND PHAGE, MODULATE EVOLUTIONARY OUTCOME

Unexpectedly, in terms of the different modes and targets of resistance for the different antibiotics, the time‐invariant rifampicin environment resulted in an increased probability of low‐level resistance to nalidixic acid and spectinomycin, specifically in the absence of phage (Fig. 3A). When comparing control and time‐invariant rifampicin environments with and without phage, nalidixic acid‐resistant clones only occurred in time‐invariant rifampicin environments without phage (hence, their prevalence between these environments cannot be compared using logistic regression). Low‐level spectinomycin resistance, in turn, occurred in a small subset of the populations also in the control environments but was extremely prevalent in the time‐invariant rifampicin environment without phage (time‐invariant rifampicin environment vs. control, *P* = 0.039; phage presence, *P* < 0.001; interaction, *P* = 0.002; for full results, see Table S11). Moreover, low‐level spectinomycin resistance was much more prevalent in time‐dependent environments in the absence of phage compared to the time‐invariant spectinomycin environment. We hypothesized that both observations could result from pleiotropic effects of mutations selected by rifampicin (Jin and Gross 1989; Balbontin et al. 2021), with either mutational targets being altered or the pleiotropic effect being modulated by the presence of phage. In line with the latter explanation, we found that six out of eight clones containing *rpoB* mutations (producing rifampicin resistance) and unexposed to the phage displayed low‐level resistance to spectinomycin. Conversely, only four out of 17 clones with *rpoB* mutations in the presence of phage displayed resistance to spectinomycin. We also found a similar pattern of antimicrobial cross‐resistance depending on phage resistance for two other genes: the stringent response gene *spoT* that was mutated across experimental treatments and the previously identified spectinomycin‐selected gene *nadR* (Fig. 3B). Consequently, clones containing mutations in either *nadR*, *rpoB*, or *spoT* had a high likelihood of exhibiting a low‐level spectinomycin resistance phenotype conditioned on occurring in a phage susceptible genomic background (ANOVA for binomial generalized linear model: presence of mutations in *nadR*, *rpoB*, or *spoT*, *χ* ^2^_1,233_ = 1.90, *P* = 0.17; phage resistance, *χ* ^2^_1,232_ = 25.7, *P* < 0.001; presence of mutations in *nadR*, *rpoB*, or *spoT* × phage resistance, *χ* ^2^_1,231_ = 7.01, *P* = 0.008).

Furthermore, we found that all *mreC* mutants (selected by rifampicin) as well as a single *mreD* mutant displayed low‐level multidrug resistance. Two of three *mreC* mutants (containing the same frameshift mutation, Glu291fs) and the *mreD* mutant were resistant to all three drugs, whereas a single *mreC* mutant (Val46Gly) was resistant to both nalidixic acid and spectinomycin but remained susceptible to rifampicin. Mutations in *mreC* and *mreD*, whose products work in concert to determine cell shape and elongation, did not occur in the presence of phage. In turn, mutations in *marR* selected by rifampicin that occurred only in the presence of phage also resulted in nalidixic acid resistance. These mutations could therefore explain the increased probability of nalidixic acid resistance in the presence of rifampicin. In addition, we found that phage resistance, associated, in particular, with mutations in *fabR*, *galU*, and ECB RS18465, had a strong fitness cost as indicated by reduced bacterial growth after 24‐hour culture (88.8% growth of phage sensitive clones) in the absence of the phage or antimicrobial compounds (ANOVA for linear model: phage susceptibility, *F*_1,898_ = 84.1, *P* < 0.001) (Fig. 3C). Together these observations suggest that nalidixic acid and spectinomycin resistance dynamics were to a large extent driven by the other two selective agents (rifampicin and phage) imposing stronger selection through the following three mechanisms: (i) cross‐resistance (i.e., pleiotropic) mutations selected in the presence of rifampicin; (ii) the effect of phage on the strength of selection for antimicrobial resistance and the targets of antimicrobial resistance mutations; and (iii) the loss of the antimicrobial resistance phenotype of specific mutations in a phage‐resistant background. Cases (ii) and (iii) may be related to the strong fitness‐impairing consequence of phage resistance.

### MODIFYING EFFECTS OF STRONG SELECTIVE ENVIRONMENTS LARGELY EXPLAIN DIFFERENCES BETWEEN TIME‐DEPENDENT ENVIRONMENTS

The evolutionary dynamics described above largely determined differences in low‐level resistance levels between the time‐dependent environments. First, rifampicin that was the strongest selective antimicrobial compound caused resistance to occur as a function of exposure epoch mainly by selecting for *rpoB* mutations followed by negative selection post exposure (Figs. 4A and S8B; rifampicin exposure epoch, *P* = 0.074; for full results, see Table S11). Second, nalidixic acid resistance occurred at a much lower level in general (Figs. 4B,C and S8A). It occurred as a function of rifampicin exposure in the absence of phage where selection by nalidixic acid was weak and rifampicin was selected for low levels of nalidixic acid cross‐resistance by *mreC* mutations (Figs. 4B and S8A; nalidixic acid exposure epoch, *P* = 0.48; rifampicin exposure epoch, *P* = 0.003; for full results, see Table S12). In the presence of phage, however, nalidixic acid resistance was more strongly driven by nalidixic acid exposure selecting for *acrR* and ECB RS03400 mutations (Figs. 2, 4, and S8B; nalidixic acid exposure epoch, *P* = 0.007; rifampicin exposure epoch, *P* = 0.22; for full results, see Table S13). This is consistent with the bacterial cells experiencing stronger selection for nalidixic acid resistance in the presence of both phage and nalidixic acid compared to nalidixic acid alone. Finally, in line with cross‐selection by rifampicin, spectinomycin resistance level was influenced by both the spectinomycin and rifampicin exposure epochs, although being mainly determined by the presence of phage (Figs. 3B and S8C; spectinomycin exposure epoch, *P* = 0.012; rifampicin exposure epoch, *P* < 0.001; phage presence, *P* < 0.001; for full results, see Table S14).

A high prevalence of low‐level spectinomycin resistance in the absence of phage accounted for a large proportion of positive resistance signals in the phenotypic dataset. Because of this, low‐level multidrug resistance (here referring to resistance to multiple antimicrobial compounds and excluding resistance to the phage) was more likely to occur in the absence of phage despite the phage exacerbating selection for rifampicin and nalidixic acid resistance in the time‐invariant single‐drug environments. As nalidixic acid selection was weak, most cases of multidrug resistance were cases of rifampicin‐spectinomycin cross‐resistance. Notably, three out of five among the sequenced clones displaying resistance to all three agents contained mutations in the cell shape determining genes *mreC* and *mreD*. Although rifampicin resistance levels began to decay after the rifampicin exposure epoch, likely owing to a fitness cost of rifampicin resistance (Jin and Gross 1989; Balbontin et al. 2021), the effect was too weak to introduce a clear history dependence effect on low‐level multidrug resistance levels. Therefore, differences in the evolutionary outcome between the time‐dependent environments were mostly accounted for by the following factors: differences in selection strength between the agents; pleiotropic effects of rifampicin resistance; and modifying effects of phage exposure on antimicrobial resistance evolution.

### INFERENCE OF ENVIRONMENTAL PAST AND PREDICTABILITY OF EVOLUTIONARY FUTURE ARE STRONGEST FOR DRIVER SELECTIVE ENVIRONMENTS

The strong selective environments (rifampicin and phage) largely accounting for the low‐level multidrug resistance landscape, correspondingly, exhibit the strongest predictive power regarding the past drug exposure and future resistance outcome of the bacterial populations (Fig. 5A). As expected based on the strong fitness consequence of phage resistance, machine learning (random forest) models were able to predict past phage exposure with high accuracy based on growth data from end‐point clones in the absence or presence of different levels of the experimental antimicrobials. The same data could also be used to train a model to precisely predict the rifampicin exposure epoch, consistent with rifampicin resistance levels both decaying after exposure and influencing the overall resistance phenotypes. Conversely, in line with expectations, high‐accuracy predictive models could not be constructed for the exposure epoch of the weak selective agent nalidixic acid or cross‐selected agent spectinomycin. These factors were also seen as modest predictive power of models predicting the full antimicrobial exposure sequence (i.e., environmental past). Therefore, the ability to infer the past from the current phenotypic state is increased by high selection coefficients and strong fitness effects of resistances and can be obscured by low selection coefficients and pleiotropy.

We further quantified the mutual information between the environmental past (antibiotic/phage exposure sequence) and end‐point phenotype (resistance to each antibiotic and phage) (Fig. 5B). A large value of mutual information between an environment and a phenotype indicates that knowing one substantially removes uncertainty about the other. Phage and spectinomycin exhibited strong mutual information (0.27 [SD 0.03]) and adding the detail of the antimicrobial exposure order further increased it to 0.51 (SD 0.03). For nalidixic acid and rifampicin, knowing whether the exposure history had phage or not carried little information. However, for both compounds, increasing the detail of the antimicrobial exposure order carried information. In contrast to the random forest modeling results, we noticed that the epoch of exposure to rifampicin did not greatly reduce uncertainty for the individual end‐point resistance states. This is due to the random forest model exploiting both population and clone data at multiple MIC values—beyond binary (quantitative OD value instead of 0 for susceptible and 1 for resistant)—as well as using the joint phenotype, respect to all compounds, as its basis for predicting (i.e. classifying) the past exposure environment.

Together these results are consistent with the previously reported strongest environment‐phenotype links, namely, the relationship between rifampicin and phage exposures and corresponding resistances, and the (inverse) relationship between phage presence and low‐level spectinomycin resistance. Therefore, establishing the relationship between driver environments (i.e., strong selective agents; here, rifampicin and phage) and phenotypic states is critical for understanding and predicting evolution along time‐dependent fitness seascapes.

## Discussion

Inspecting differences in evolutionary dynamics between time‐invariant and time‐dependent fitness seascapes, we found that specific environments drove environment‐phenotype links. These driver environments (here, one subinhibitory antimicrobial, rifampicin, and phage) were characterized by strong selective pressure on the target adaptive trait (rifampicin and phage resistance at the phenotypic and genetic levels) and pleiotropic effects on off‐target traits (growth ability and susceptibility to the other antibacterial agents, along with related mutational changes). Furthermore, we showed that establishing the driver environments and their phenotypic consequences is key for predicting evolution along fitness seascapes. Our data are also consistent with the order of the driver agents strongly influencing the evolutionary outcome in time‐dependent fitness seascapes compared to the order in general. These findings expand previous findings showing the importance of a limited number of key drivers in multistressor environments into a temporal setting (Brennan 2015; Boyd et al. 2016; Brennan et al. 2017). The practical importance of this finding is stressed by the fact that time‐dependent fitness seascapes in real‐life scenarios are highly unlikely to contain a set of environments identical in terms of the strength of selection and the strength of epistatic and pleiotropic effects. Therefore, any effort to construct general laws and models for evolution along fitness seascapes should account for temporal or environmental differences in the strengths of these factors.

The driver agents may partly be accounted for by higher selection pressure. In our study, nalidixic acid that exerted the smallest selective effect also has the smallest effect on the growth rate (*r*) of the bacteria relative to the antibiotic‐free environment (0.88). In contrast, of the driver agents, rifampicin has a strong effect on bacterial growth at the experimental concentration (0.46), and the phage is virulent, lysing all susceptible cells and therefore imposing strong selective pressure. Intriguingly, however, spectinomycin has the strongest growth effect (0.36) but did not drive the system similarly to rifampicin and the phage. This suggests that also other factors contribute to an agent driving evolution in a multidrug setup, such as pleiotropic effects of resistance mutations previously documented for rifampicin (Jin and Gross 1989; Balbontin et al. 2021). It should also be noted that growth in the antibiotic environment is not the only factor determining selection pressure but it is also affected by factors such as the fitness effects of resistance mutations.

Although our experimental setting using sub‐MIC selection (0.5 × MIC) did not mimic therapeutic antimicrobial levels, our findings have a number of potential implications concerning the global antimicrobial resistance crisis. First, sub‐MIC levels of antimicrobials occur in many human‐impacted environments such as wastewater and agricultural environments, as well as in the concentration gradients within the tissues of medicated humans, production animals, and pets. In these conditions, depending on the antimicrobial compound, even very low concentrations can increase the fitness of resistant cells above that of susceptible cells, causing positive selection for resistance (Gullberg et al. 2011). Because of weaker selection compared to high antibiotic levels, mutations producing resistance in such conditions cannot afford to be coupled with strong fitness costs (Gullberg et al. 2011). This can lead low‐antibiotic‐level environments to facilitate the stepwise evolution of high‐resistance, low‐fitness‐cost mutants particularly problematic to remove if they spread among humans or production animals as pathogens (including opportunistic pathogens) (Baym et al. 2016). Our findings demonstrate that particular driver agents (here rifampicin) can create low levels of resistance against multiple drugs even at sub‐MICs, a phenomenon (pervasive pleiotropy causing cross‐resistance or collateral susceptibility) studied previously mainly for high antimicrobial levels (Cairns et al. 2017; Rosenkilde et al. 2019; Liu et al. 2020). This finding expands the scope of the potential undesired consequences of environmental antimicrobial residues. Such environments are also highly likely to experience residues of different antimicrobials over time, creating fitness seascapes similar to those included in our study setup. Under such conditions, it may be important to establish the historical exposure of the environment to particular driver agents as part of risk estimation for subsequent antimicrobial contamination.

Second, the driver agents modified the interplay between selection and the epoch length by exacerbating selection and thus partially removing the desired filtering effect of epochs to resistance evolution. As the driver agents affected resistance evolution to both directions, assessing their overall impact for a specific therapy requires experimentation. Clearly, identifying and testing the impact of such driver agents has potential for therapy optimization and their efficient usage should be studied further using eco‐ evolutionary control theory (Lässig and Mustonen 2020). Intriguingly, driver agents do not necessarily need to be antimicrobials; for instance, the effects of stress environments can be further modulated by inhibiting global regulators (Jarosz and Lindquist 2010).

Interestingly, it was recently demonstrated that cellular hysteresis whereby transgenerational changes in cellular physiology induced by one drug alter the bacterial response to a second drug can influence bacterial evolutionary trajectories in alternating drug protocols, particularly with rapid (every 12 or 24 hours) switching (Roemhild et al. 2018). It is conceivable that cellular hysteresis could have contributed to the evolutionary outcomes in this study. Because the number of putative resistance mutations accumulated over exposure to three drugs and phage tended to be low (two on average), pleiotropic effects of previously accumulated mutations are likely to explain only a fraction of the variance in evolutionary trajectories between time‐dependent protocols. An interesting avenue of future exploration is whether the driver environments are also more likely to induce cellular hysteresis. This could contribute to particular drugs functioning as driver agents in multidrug systems.

Our study also lends general insights into ecological resilience critical to understand owing to the climate and biodiversity crisis. Based on our study findings, when organisms evolve in temporally changing environments, particular environments (especially stressors) are likely to play a pivotal role in facilitating or obstructing multi‐environmental adaptation relative to the sequence of environments alone (Brennan 2015; Boyd et al. 2016; Brennan et al. 2017). Identifying such key environmental conditions and their off‐target effects may be critical in fields such as nature conservation for preventing population or community collapse and for enhancing their biological resilience. This study and a recent study with a microbial system (Hiltunen et al. 2018) suggest that strong off‐target effects of adaptations to particular environments may be commonplace. Therefore, a failure to incorporate them in ecological and evolutionary predictions can severely restrict the efficacy of interventions based on them.

Finally, fitness seascapes have been studied both experimentally and theoretically much less than their static counterparts. A reason behind this gap is the apparent complexities involved—it is much harder to convince oneself that a particular fitness seascape constitutes a minimal model worth studying at depth compared to the canonical models of static landscapes familiar from textbooks (Gillespie 1992). However, our results show that experimental work on the topic is both feasible and informative for future theoretical work. Indeed, the possibility of simplifying evolution under complex seascapes by describing them in terms of a few driver agents looks theoretically viable, although the strength of selection and duration of exposure are likely to considerably impact tractability.

## AUTHOR CONTRIBUTIONS

JC, TH, and VM conceptualized the idea of the study. JC and VM performed supervision. JC, TM, FB, and VM curated the data. JC, VM, FB, and TM performed formal analysis, visualization, and investigation. JC wrote the original draft. All authors reviewed and edited the manuscript.

## CONFLICT OF INTEREST

The authors declare no conflict of interest.

## DATA ARCHIVING

Raw sequence read data have been deposited in the NCBI SRA under the accession PRJNA768654. All preprocessed data used for the downstream analyses and figures will be available via Dryad. https://doi.org/10.5061/dryad.zgmsbccdn

Associate Editor: Dr. Katrina Lythgoe

## Supporting information

Supplementary Figure S1. Confirmation of REL607‐like Ara+ reversion mutation. Related to Figure 2, Figure 3, Figure 4, and Figure 5.
Supplementary Figure S2. Malthusian parameters for the two ancestral strains in the serial passage experiment showing lack of difference in initial fitness.
Supplementary Figure S3. Dose response curve for REL606 carrying capacity in the experimental antimicrobials.
Supplementary Figure S4. Intrinsic growth rate (r) of ancestral REL606 in the experimental antimicrobial concentrations.
Supplementary Figure S5. Effect of well plate location on background optical density (OD) value at 600 nm obtained from the Tecan Infinite M200 device used to quantify the growth of end‐point clones from serial passage experiment.
Supplementary Figure S6. Distribution of OD data for clonal lines after removing well‐specific background OD values. Related to Figure 2, Figure 3, Figure 4, and Figure 5.
Supplementary Figure S7. Ability of clones isolated from the end‐point of evolutionary experiment to grow at different concentrations of the experimental antimicrobials.
Supplementary Figure S8. Antimicrobial resistance spectra in different time‐dependent regimes. Related to Figure 2, Figure 3, Figure 4, and Figure 5.
Supplementary Table S1. Related to Figure 2, Figure 3, Figure 4, and Figure 5.
Supplementary Table S2. Related to Figure 2, Figure 3, Figure 4, and Figure 5.
Supplementary Table S3. Related to Figure 2, Figure 3, Figure 4, and Figure 5.
Supplementary Table S4. Related to Figure 2, Figure 3, Figure 4, and Figure 5.
Supplementary Table S5. Related to Figure 2, Figure 3, Figure 4, and Figure 5.
Supplementary Table S6. Related to Figure 2, Figure 3, Figure 4, and Figure 5.
Supplementary Table S7. Related to Figure 2, Figure 3, Figure 4, and Figure 5.
Supplementary Table S8. Related to Figure 2, Figure 3, Figure 4, and Figure 5.
Supplementary Table S9. Related to Figure 2, Figure 3, Figure 4, and Figure 5.
Supplementary Table S10. Related to Figure 2, Figure 3, Figure 4, and Figure 5.
Supplementary Table S11. Related to Figure 2, Figure 3, Figure 4, and Figure 5.
Supplementary Table S12. Related to Figure 2, Figure 3, Figure 4, and Figure 5.
Supplementary Table S13. Related to Figure 2, Figure 3, Figure 4, and Figure 5.
Supplementary Table S14. Related to Figure 2, Figure 3, Figure 4, and Figure 5.
