# Supplementary material for: Strong selective environments determine evolutionary outcome in time‐dependent fitness seascapes
Source: Evol Lett. 2022 May 26;6(3):266–79. doi: 10.1002/evl3.284 (PMC9233173; doi:10.1002/evl3.284)
Supplement: Supplementary file 1 — Supplementary Figure S1. Confirmation of REL607‐like Ara+ reversion mutation. Related to Figure 2, Figure 3, Figure 4, and Figure 5. Supplementary Figure S2. Malthusian parameters for the two ancestral strains in the serial passage experiment showing lack of difference in initial fitness. Supplementary Figure S3. Dose response curve for REL606 carrying capacity in the experimental antimicrobials. Supplementary Figure S4. Intrinsic growth rate (r) of ancestral REL606 in the experimental antimicrobial concentrations. Supplementary Figure S5. Effect of well plate location on background optical density (OD) value at 600 nm obtained from the Tecan Infinite M200 device used to quantify the growth of end‐point clones from serial passage experiment. Supplementary Figure S6. Distribution of OD data for clonal lines after removing well‐specific background OD values. Related to Figure 2, Figure 3, Figure 4, and Figure 5. Supplementary Figure S7. Ability of clones isolated from the end‐point of evolutionary experiment to grow at different concentrations of the experimental antimicrobials. Supplementary Figure S8. Antimicrobial resistance spectra in different time‐dependent regimes. Related to Figure 2, Figure 3, Figure 4, and Figure 5. Supplementary Table S1. Related to Figure 2, Figure 3, Figure 4, and Figure 5. Supplementary Table S2. Related to Figure 2, Figure 3, Figure 4, and Figure 5. Supplementary Table S3. Related to Figure 2, Figure 3, Figure 4, and Figure 5. Supplementary Table S4. Related to Figure 2, Figure 3, Figure 4, and Figure 5. Supplementary Table S5. Related to Figure 2, Figure 3, Figure 4, and Figure 5. Supplementary Table S6. Related to Figure 2, Figure 3, Figure 4, and Figure 5. Supplementary Table S7. Related to Figure 2, Figure 3, Figure 4, and Figure 5. Supplementary Table S8. Related to Figure 2, Figure 3, Figure 4, and Figure 5. Supplementary Table S9. Related to Figure 2, Figure 3, Figure 4, and Figure 5. Supplementary Table S10. Related to Figure 2, [file EVL3-6-266-s001.pdf]

Supplementary Information for

Strong selective environments determine evolutionary outcome in time-dependent fitness seascapes

Johannes Cairns, Florian Borse, Tommi Mononen, Teppo Hiltunen, and Ville Mustonen

Correspondence to: johannes.cairns@helsinki.fi / v.mustonen@helsinki.fi / teppo.hiltunen@utu.fi

This PDF file includes:

Supplementary methods
- Confirmation of REL607-like Ara$^+$ reversion mutation
- Competition assay for ancestral strains: *E. coli* REL606 and REL607-like Ara$^+$ reversion mutant
- Details on serial passage experiment
- Determining minimum inhibitory concentrations for antimicrobials for ancestral *E. coli* strain
- Measuring antimicrobial resistance phenotypes
- Measuring phage presence and resistance phenotypes
- Plaque assay and generation of T4 phage stock
- Details on phenotyping experimental populations over time
- Details on phenotyping clones isolated from experimental end-point
- Details on extraction of DNA from clones isolated from experimental end-point
- Genome alignment, variant calling, and annotation
- Details on machine learning analysis

Supplementary results
- Details on potential molecular targets of resistance

Figures S1 to S8
Tables S1 to S14

References

Supplementary methods

Confirmation of REL607-like Ara+ reversion mutation

The revertant produced for this study contains the same ara mutation (92G / GGC; REL606 ancestor: 92D / GAC) as the REL607 strain used in the *E. coli* Long-term Experimental Evolution project in the Lenski lab (barricklab.org/twiki/bin/view/Lab/ProtocolsAraMarker, accessed 2018-05-15). The *E. coli* B strain REL606 was acquired from the *E. coli* Genetic Resources at Yale CGSC, The Coli Genetic Stock Center (REL606 CGSC#: 12149). A liquid culture of the strain was streak-plated on Lysogeny Broth (LB) agar medium, and five clones were isolated by culturing five separate colonies in DM1000 medium and freeze-storage with glycerol at –80 °C. All culturing steps were performed for 24 h at 37 °C (liquid cultures with constant rotation at 150 r.p.m.), unless specified otherwise. To select for REL607-like Ara+ revertants derived from REL606 (Ara–), a colony of each of the five clones was cultured in six replicates of 10 mL of DM1000 medium, spun down to concentrate cells (10^9 cells), and plated on MA agar medium, followed by culturing for 48 h (personal correspondence with Richard Lenski, 2018-04-27). Ara+ colonies were re-streaked on MA plates, confirmed as Ara+ based on white color when cultured on TA agar medium, and an individual Ara+ colony from each of the five lines was isolated by culturing in DM1000 medium and freeze-storage with glycerol at –80 °C.

One REL606 colony and its derived Ara+ revertant were confirmed by Sanger sequencing. For this, colony PCR was performed using the following primers for the *ara* operon (Meyer et al. 2015): REL256 (coordinates for REL606: 70,660–70,783) 5'-CCGATACGCTCATGGGCTTGTTTA-3' and REL257 (coordinates: 71,177–71,154) 5'-CTGCCCAGGCCGTTGCGACTCTAT-3' . To release DNA for the PCR reaction, 2–3 colonies (Ø 2 mm) from plates containing 24–48 h pure cultures were transferred to 50 μL of 20 mM NaOH, boiled for 10 min at 100 ◦C, spun down, vortexed, and spun down again. PCR was performed with 2 μL of the resultant solution for use as template DNA, 1.25 U of Taq DNA Polymerase (New England Biolabs, Ipswitch, MA, USA), 0.2 μM each of the *ara* primers,

and 200 μM of dNTPs in a final volume of 50 μL of 1 × Standard Taq Reaction Buffer. The cycling conditions were as follows: 95 °C for 5 min to enhance cell lysis, and 30 cycles of 95 °C for 30 s, 56 °C for 30 s and 72 °C for 1 min, with a final extension at 72 °C for 5 min. The PCR products were Sanger sequenced by a third party (Institute of Biotechnology, University of Helsinki, Finland) using in-house protocols. The sequences were assembled from Sanger sequencing chromatograms using Pregap4 and Gap4 in the Staden Package (Staden et al. 2000), and aligned with ClustalW using MEGA7 (Kumar et al. 2016) (Fig. S1).

Competition assay for ancestral strains: *E. coli* REL606 and REL607-like Ara+ reversion mutant

To ascertain that there was no initial difference in fitness between the two ancestral strains, a competition assay was carried out following a previously established protocol (barricklab.org/twiki/bin/view/Lab/ProceduresLongTermCompetitions, accessed 2018-05-16). For the assay, the strains were revived by culturing 2 μL of freeze-stored clonal culture in 5 mL LB liquid medium overnight at 37 °C with constant rotation at 120 r.p.m. Subsequent culturing steps were performed in DM1000 medium. Before starting the competition assay, the strains were mixed together at 200-fold dilutions (100-fold overall dilution in cell number), and this mix was immediately dilution plated on TA agar to determine the initial frequencies of the strains. Subsequently, 50 μL of each of the strains was added to 10 mL medium, with 12 replicates, followed by culturing at 37 °C, and serially diluting (1 % volume) each day for a total of three days. This allows relative fitness to be determined with a precision of ± 1 % (95 % confidence intervals). Upon completion of the assay, the cultures were immediately dilution plated on TA agar to determine the final frequencies of the strains.

The Malthusian parameters for the two strains are given by $M_{REL606} = \frac{N_{REL606}(f)}{N_{REL606}(i)} = \frac{PC_{REL606}(f) \cdot DF}{PC_{REL606}(i)}$ and $M_{REL607-like} = \frac{N_{REL607-like}(f)}{N_{REL607-like}(i)} = \frac{PC_{REL607-like}(f) \cdot DF}{PC_{REL607-like}(i)}$, where *N* is the cell number, *PC* the plate count on TA agar, *DF* the dilution factor of all transfers combined, and *i* and *f* the initial and final time points,

respectively. A difference between the logarithm of the Malthusian parameters (ratio ≠ 1) for the two strains (paired for each replicate) indicates a difference in fitness. The result of the competition assay (one of 12 replicates was lost due to technical error, *i.e.* $N = 11$) supports the absence of a difference in fitness between the two ancestral strains (Fig. S2).

Fig. S2 was created using the method by (Ho et al. 2019) (tool available at http://www.estimationstats.com, accessed 2019-07-19). The paired mean difference between log(M_{REL606}) and log($M_{REL607\text{–}like}$) is -0.0056 [95.0 % CI -0.0488, 0.0418]. The two-sided *P*-value of the Wilcoxon test is 0.594. The effect sizes and CIs are reported above as: effect size [*CI width lower bound; upper bound*]. 5000 bootstrap samples were taken; the confidence interval is bias-corrected and accelerated. The *P*-value reported is the likelihood of observing the effect size if the null hypothesis of zero difference is true.

Details on serial passage experiment

The experiment was started by culturing the REL606 and REL607-like strains in DM1000 for 24 h to obtain 2×10^9 cells mL^{-1}. One colony from each plated strain was added to 2 × 5 mL of DM1000, and from this solution, approximately 10^6 bacterial cells (50 μL) of the corresponding strain were pipetted to the wells of the deep well plates containing 500 μL of DM1000 with 0.5 × MIC of the appropriate antimicrobial. In addition, 5×10^5 plaque forming units (PFU) of the T4 phage (13 μL from phage stock containing 3.9×10^9 PFU mL^{-1}) were pipetted to phage treatment wells. Deep well plates were cultured at 37 °C with constant shaking at approx. 120 r.p.m. Serial passage was performed every 24 h by transferring 10 μL (2 % v/v) from each well to a new well containing fresh medium using a multichannel pipette. To store populations in suspended animation, after the first 48 h and subsequently every 96 h in the experiment, all deep well plates were freeze-stored at –20 °C by mixing in 250 μL of sterile 85 % glycerol in each well and covering with a foil seal.

Determining minimum inhibitory concentrations for antimicrobials for ancestral *E. coli* strain

The MIC values for the experimental antimicrobials were determined for the REL606 strain in the experimental conditions using the microdilution method. Instead of 2-fold dilutions typically used, the experiment was performed at even concentration intervals in a total interval determined based on expected MIC values: for nalidixic acid, 1–5 μg mL^{-1} (expected MIC of μg mL^{-1} for REL606 based on (Harmand et al. 2018); for rifampicin, 1–5 μg mL^{-1} (expected MIC of 2.5 μg mL^{-1} for *E. coli* B (Rodriguez-Verdugo et al. 2013)); for spectinomycin, 10–20 μg mL^{-1} (expected value of 10–20 μg mL^{-1} for *E. coli* B (Anderson 1969)). The experiment was performed by adding 10^6 cells (1/2000 dilution of overnight culture of REL606 in DM1000 medium) to honeycomb wells containing DM1000 medium, with each well containing a different concentration of the antimicrobial in question according to the gradients specified above. This cell number is expected to be lower than the spontaneous frequency of resistance mutations, indicating that any observed growth represents the intrinsic resistance level of the ancestral strain rather than that of derived mutants emerging during the test. Culturing was performed for 24 h at 37 ºC using the Bioscreen C well-plate reader (Labsystems, Helsinki, Finland) that measures optical density (OD) at 420–580 nm with a wideband filter at 5 min intervals.

The MIC value was interpreted as the smallest concentration preventing growth, which was 1.0, 2.6 and 8.0 μg mL^{-1} (experimental concentrations of 0.5 × MIC thereby being 0.5, 1.3 and 4.0 μg mL^{-1}) for nalidixic acid, rifampicin, and spectinomycin, respectively (Fig. S3). The dose response curves in Fig. S3 show that the antimicrobial level where the carrying capacity begins to decrease is lower than the concentration (0.5 × MIC) used in the experiment. This indicates that differences in effective population size caused by the antimicrobials do not account for differences in evolutionary dynamics between the treatments in the absence of evolution altering the population size response to the antimicrobials.

Measuring antimicrobial resistance phenotypes

To quantify the evolution of antimicrobial resistance over time, every 2 days in the 48-day evolutionary experiment, 2 % (10 μL) of the old culture was transferred, using a 96-pin replicator, to a large petri dish containing lysogeny broth (LB) agar supplemented with one of the three antimicrobials at a selective concentration (> 1 × MIC), including an antimicrobial-free plate to control for bacterial extinction. After culturing for 24 h at 37 ºC, growth on the plates was quantified on a binary scale (0 = no growth; 1 = growth). Therefore, for this metric, growth indicates the detectability of antimicrobial resistance in a minimum of 2 % of the bacterial population. Notably, we cross-phenotyped all populations from all treatments and time points against all antimicrobials. Since the temporal tracking of resistance evolution employed a coarse measure diverging from experimental conditions (solid medium vs. liquid medium; higher antimicrobial level compared to selective concentration of 0.5 × MIC; phenotype based on fraction of heterogeneous population), we isolated individual clonal lineages from the experimental end-point and phenotyped them more precisely. Clones were isolated by streak-plating inoculum from the population on an LB agar plate, culturing overnight, and picking an individual colony, followed by propagation in liquid medium. The clones were phenotyped by culturing a small initial number of cells for 24 h in DM1000 liquid medium without antimicrobials or supplemented with one of the three antimicrobials at a range of multiplicities of the MIC value of the ancestral *E. coli* strain: 1, 2, 10, or 100 × MIC. This allowed obtaining quantitative growth and antimicrobial resistance phenotypes (in units of optical density at 600 nm, OD_{600}) in the experimental conditions for isogenic lineages likely to represent the dominant *E. coli* genotype in each of the 900/928 populations that escaped extinction during the 48-day serial passage experiment. Before downstream analyses, the OD data was curated by removing background OD values specific to 96-well plate location, drug type, and drug concentration (Fig. S4), and following this, by removing (transforming to zero growth) likely false positive values based on belonging to a distinct distribution close to zero (Fig. S5). The cut-off used to remove false positives was 3 times the standard deviation of the noise distribution close to zero, representing a demarcation region between the noise distribution and true

positive value distribution. In later analyses, resistance was defined for the clones as the capacity to grow at antimicrobial concentrations exceeding the MIC of the ancestral strain, as the experimental concentration of 0.5 × MIC selected mostly for low-level resistance (Fig. S6).

Measuring phage presence and resistance phenotypes

We also measured the loss of phage from phage-containing populations during the experiment using the population level pin replicator system on agar plates containing a soft agar lawn of the ancestral *E. coli* strain, where the formation of a plaque indicates presence of phage. Moreover, we quantified the phage resistance phenotype of the end-point clones using a similar setup, with a lawn of the ancestral T4 phage instead of the bacterium. Bacterial growth on the phage lawn was taken to indicate phage resistance, which was therefore characterized as a binary trait (susceptible/resistant). Detailed phenotyping protocols are documented below.

Plaque assay and generation of T4 phage stock

The T4 phage stock for the experiment was generated based on previously established protocols (Lenski lab protocol: lenski.mmg.msu.edu/ecoli/phagepharm.html, accessed 2018-05-15; Barrick lab protocol: barricklab.org/twiki/bin/view/Lab/ProceduresPhageFarming, accessed 2018-05-18). First, a dilution series of an existing T4 stock was prepared in LB liquid medium, and 100 μL was combined with 100 μL of exponentially growing host strain (REL606). Following incubation at 37 °C for 30 min, the mixture was added to 4 mL of soft LB agar (7 g agar per litre) kept at 55–60 °C, mixed and poured on top of an LB agar plate. The plates were cultured for 24 h at 37 °C, generating phage plaques at dilutions where the plaques are not touching. Subsequently, 1 mL of overnight host culture was transferred to 4 mL of LB medium in a 50 mL flask, and a toothpick was stuck into an isolated plaque

and dropped into the host culture, which was kept at 37 °C with constant shaking at 150 r.p.m. After 6–8 h causing complete lysis of the host, 10 drops of chloroform were added and mixed to kill the remaining bacteria, and cell debris was removed by spinning down and keeping the supernatant. The phage was then titred as described above to determine phage density in the stock (as plaque forming units, or PFU, *i.e.* infectious phage particles). The lysate was stored with glycerol at –80 °C.

Details on phenotyping experimental populations over time

To track the development of antimicrobial resistance over time, the following selective concentrations were chosen for each antimicrobial for use in LB agar plates based on literature: 2 and 20 μg mL^{-1} for nalidixic acid, 50 μg mL^{-1} for rifampicin, and 50 and 75 μg mL^{-1} for spectinomycin. After each 24 h growth cycle in the experiment, the populations were transferred to selective plates, as well as plates without antimicrobials to control for the presence of bacteria, by pin-replicating the whole deep well plate onto a large petri dish. To assess the presence of phage, 200 μL of overnight culture of the ancestral REL606 strain into was added to a sterile 15 mL Eppendorf tube, followed by adding 15 mL of soft agar tempered to 55 °C in a water bath, vortexing, pouring on large petri dish containing LB agar, and pin-replication of the 24 h culture from the experiment upon solidification of soft agar. Plates were cultured for 24 h at 37 °C. Each plate was documented by photography, and the results were interpreted as negative (0), weak (1), or strong (2) bacterial growth (antimicrobial resistance) or plaque formation (phage presence), subsequently converted to binary data (2 converted to 1).

Details on phenotyping clones isolated from experimental end-point

Clones were isolated from the experimental end-point by streaking an inoculum from surviving populations (N = 900 out of 928 populations in total) on LB plates. Following culture (24 h / 37 °C), a single colony was selected and transferred to a 96 well plate containing 200 μL of DM1000. Following culture (24 h / 37 °C / 120 r.p.m.), the clonal lines were freeze-stored with 85 % glycerol at –80 °C. To

perform subsequent tests, the master well plates containing the original clones were replicated on 96 well plates containing 200 μL of DM1000. The test well plates were cultured and freeze-stored as above. To obtain quantitative growth traits (optical density at 600 nm) for different antimicrobial concentrations, a test well plate was replicated on another 96 well plate containing 200 μL of DM1000, and cultured for 24 h at 37 °C / 120 r.p.m. This results in a cell density of 2×10^9 cells mL^{-1}. The clonal cultures were diluted into deep well plates to obtain a total dilution of 1:2000, corresponding to 1×10^6 cells mL^{-1} assuming a density of 2×10^9 cells mL^{-1}. This cell count is expected to be lower than the frequency of spontaneous antimicrobial resistance mutations, indicating that any observed growth represents the (potentially evolved) resistance level of the clonal line rather than that of derived mutants emerging during the test. Approx. 2000 cells (2 μL) of each diluted clone was pipetted on another 96 well plate containing 300 μL of DM1000 with each of the experimental antimicrobials at the following concentrations: 0, 1, 2, 10, or 100 × MIC (respectively: 0, 1.0, 2.0, 10, and 100 μg mL^{-1} for nalidixic acid; 0, 2.6, 5.2, 26, and 260 μg mL^{-1} for rifampicin; and 0, 8.0, 14, 80, and 800 μg mL^{-1} for spectinomycin. Following culture for 24 h at 37 °C / 120 r.p.m., the cell density reached by each clone was quantified by measuring OD at 600 nm wavelength (Tecan Infinite M200) with a bandwidth of 9 nm and 25 measurements per well. The REL606 and REL607-like strains and blank wells containing the medium alone supplemented with the different antimicrobials (only rifampicin was shown to alter the background OD level) were used as controls in each well plate. To determine the phage resistance phenotype of the clones from the phage treatments, a work plate containing REL606 and REL607-like strains as controls was pin-replicated on an LB plate containing a top layer of soft agar with the ancestral T4 phage (see detailed protocol for plaque assay above). Following culture for 24 h at 37 °C, phage resistance was quantified for each clone as a binary trait based on the absence (0) or presence (1) of bacterial growth on the phage lawn.

Details on extraction of DNA from clones isolated from experimental end-point

DNA extraction was performed from 1 mL of overnight culture with the Qiaqen DNeasy 96 Blood & Tissue kit using a custom protocol. To release nucleic acids, the cells were transferred on S plates and spun down for 30 min at 6200 r.p.m., followed by addition of 200 μL of ATL and proteinase K solution (prepared by mixing 36 mL of ATL and 4 mL of proteinase K) to each well, vortexing for 15 s with plastic cover attached, and briefly spinning down at 3000 r.p.m. The suspension was incubated for 30 min at 56 °C, with vortexing every 10 min. Subsequently, 410 μL of AL solution was added to each well, followed by vortexing for 15 s, and spin down at 3000 r.p.m. as described above. The whole sample (approx. 620 μL) was transferred to the extraction membrane columns, followed by spinning down for 10 min at 6000 r.p.m. in room temperature (RT) with plastic cover attached and discarding the flow-through. Subsequently, 500 μL of AW1 was added, followed by spinning down at 6000 r.p.m. in RT and discarding the flow-through. The plastic cover was removed, followed by adding 500 μL of AW2, spinning down for 15 min at 6000 r.p.m. in RT, discarding the flow-through, and centrifuging empty wells for 2 min at 6000 r.p.m. in RT to remove ethanol residues. After the ethanol was allowed to evaporate for 2 min in RT, membrane columns were transferred to elution plates. Elution was performed in DNA-free grade water by incubating in 40 μL of elute for 2 min in RT, centrifuging for 2 min at 6000 r.p.m., adding another 40 μL of sterile DNA-free grade water and centrifuge immediately for 2 min at 6000 r.p.m. The extracted DNA was stored at –20 °C.

Genome alignment, variant calling, and annotation

Quality controlled FASTQ files were aligned to the reference genome (NCBI Reference Sequence NC 012967, assembly ASM1798v1) with Bowtie 2 v2.3.4 (Langmead and Salzberg 2012) using default settings. SAMtools v1.4 (Li et al. 2009) was used to convert thus obtained SAM files to BAM files, and to sort and index BAM files. Picard v2.18.10 (http://broadinstitute.github.io/picard) was used to mark duplicates and add read group information to BAM files, and following these steps, to compute genome

coverage and other alignment metrics (commands CollectAlignmentSummaryMetrics and CollectWgsMetrics). Variant calling was performed with the Genome Analysis Toolkit (GATK) v3.8 (McKenna et al. 2010), including indel realignment (commands RealignerTargetCreator and IndelRealigner), and variant calling for each clone separately with a combination of HaplotypeCaller (sample ploidy set to 1) and GenotypeGVCFs. Subsequently, the GATK commands SelectVariants and VariantFiltration were used to hard filter single-nucleotide polymorphism (SNP) and short insertion and deletion (indel) data separately, using the following criteria: for both, the absence of any variants detected in the ancestral strains (–discordance), a minimum Phred-scaled quality *P*-value normalized by allele depth (QD) of 2 to remove low-quality variants, and a maximum of 2 variants in a 20 bp window (–clusterSize 2 and –clusterWindowSize 20) to remove potential variant dense regions; for SNP data, a maximum Phred scaled *P*-value from the Fisher's Exact Test to estimate strand bias (FS) of 60, a minimum root mean square mapping quality over all the reads at the site (MQ) of 40, a minimum value of –12.5 from the Rank Sum Test mapping qualities comparing the reads supporting the reference allele and the alternate allele (MQRankSum), and a minimum value of –8.0 from the Rank Sum Test for site position within reads (ReadPosRankSum); for indel data, maximum FS of 200, and minimum ReadPosRankSum of –20.0. Filtered genomic variant data was annotated against the reference genome with SnpEff v4.3i (Cingolani et al. 2012). The ancestral REL606 strain used in the experiment had no variants relative to the reference genome, while the REL607-like strain was confirmed to have the *araA* mutation as well as one additional variant, a stop-lost variant in the pseudogene ECB RS25640 encoding a hypothetical protein.

Details on machine learning analysis

Before analyses, control treatments were excluded and rare features were removed based on min. 90 % of values being zero, and the data was standardized by converting each value into a *Z*-score (subtracting each sample's mean and dividing by the sample's standard deviation). The package *splitstackshape* (Mahto 2019) was used to assign 80 % of each antimicrobial history into a training set and the rest 20 %

into a test set. This was followed by random forest classification using the function randomForest implementing the Breiman's random forest algorithm, with the options importance = TRUE and proximities = TRUE. The model was evaluated on the test set using the predict function, and the observed and predicted data were used to produce a confusion matrix with the confusionMatrix function in the *caret* package (Kuhn 2016). To avoid prediction bias due to a small test set size, this procedure was iterated 100 times, and the resulting confusion matrices were averaged over the iterations to produce one aggregate confusion matrix. The predictability of each estimated factor was interpreted as the weight of the diagonal (observed value matches predicted value) in the confusion matrix.

Supplementary results

Details on potential molecular targets of resistance

Nalidixic acid resistance: Nalidixic acid exposure was associated with a small number of nonsynonymous mutations overall (median of 0 mutations in time-invariant single agent environment in the absence of phage, equivalent to control environment) as well as a relatively small number of recurrent mutational targets occurring only in a small proportion of the isolates (Fig. 2G). This is consistent with major clones lacking resistance in time-invariant nalidixic acid environment in the absence of phage (Fig. 2C). These targets include *acrR* (encoding HTH-type transcriptional regulator), *rfaQ* (lipopolysaccharide core heptosyltransferase) and ECB RS03400 (putative phosphoglucomutase). Among these genes, *acrR* has been previously implicated in quinolone resistance (Schneiders et al. 2003), while the product function (LPS biosynthesis) and previous findings suggest that *rfaQ* (Girgis et al. 2009) and phosphoglucomutase (Paterson et al. 2009; Correia et al. 2014) may be associated with resistance to both quinolone and phage.

Rifampicin resistance: The vast majority of clones from the time-invariant single agent environment had nonsynonymous mutations (median of 1.5 mutations in the absence of phage) in the gene *rpoB* (β subunit of RNA polymerase) known to produce rifampicin resistance (Mariam et al. 2004), and the presence of mutations in this gene was almost exclusively associated with a resistance phenotype (Fig. 2G). In addition, five other genes (*galU*, *infB*, *marR*, *mreC* and *mrdB*) were recurrently mutated only in the presence of rifampicin. Among these, *marR* is related to drug efflux (Grove 2013); *mreC* and *mrdB* are both related to cell shape, with *mreC* mutations having been previously associated with rifampicin exposure (Gliniewicz et al. 2015); and mutations in the essential gene *infB* (translation initiation factor 2) have been previously implicated in rifampicin resistance (Huseby et al. 2020) and compensating for the fitness cost of antimicrobial resistance mutations (Zorzet et al. 2010). The gene

galU is involved in LPS biosynthesis and has been previously implicated in phage resistance (Le et al. 2014), and consistent with this, was only hit in the presence of phage in this study.

Spectinomycin resistance: The time-invariant spectinomycin (aminoglycoside class) environment, either in the presence or absence of phage, led to higher mean spectinomycin resistance prevalence among end-point clones compared to the antimicrobial-free control and time-invariant combination environments, although resistance increased markedly only in the time-dependent environment in the absence of phage (Fig. 2E; antimicrobial regime, $P < 0.001$; for full results, see Table S5). We were unable to obtain robust population-level time series data for spectinomycin, as the majority of populations in all test conditions (time points and spectinomycin concentrations) gave a positive result with the pin replication on agar plate method, masking any potential differences between experimental treatments. There are a number of factors that could enable population growth in our selective conditions, resulting in a positive resistance signal despite all or most of the cells remaining sensitive. Resistance may occur only in a subset of the population owing to a low selection coefficient, reversible amplifications (Andersson et al. 2019; Nicoloff et al. 2019), or plastic gene regulatory changes induced by antimicrobial stress (*e.g.* SOS or stringent response) (Poole 2012; Strugeon et al. 2016). Three genes, *nadR* (transcriptional regulator), *trkH* (potassium uptake protein) and ECB RS09520 (putative carboxyl-terminal processing protease), were recurrently mutated specifically in the presence of spectinomycin (median of 1 nonsynonymous mutation in time-invariant single agent environment), with only *trkH* reaching high (close to 0.5) frequencies (Fig. 2G). Among these, *trkH* has been previously implicated in aminoglycoside resistance (Lazar et al. 2013; Oz et al. 2014), while *nadR* (Zhang et al. 2018) and proteases (Culp and Wright 2017) can, among other functions, be related to the bacterial stress response. The observations that resistance development was difficult to distinguish in the time-series data and that two of the three potential molecular resistance mechanisms detected were linked to generic stress response rather than being specific to the antibiotic agent could be indications of weak selection pressure for resistance in the time-invariant spectinomycin environment. As much higher resistance levels occur in a subset of the time-dependent environments, with a shorter

spectinomycin selective window, these are expected to arise from the other agents (see results section ”Pleiotropic and fitness effects of strong selective agents, rifampicin and phage, modulate evolutionary outcome” in main text).

Phage resistance: Phage exposure caused recurrent mutations in several genomic targets almost exclusively associated with phage resistant phenotypes, with most targets exhibiting low to moderate (max. 0.5) prevalence among isolates (Fig. 2G). This indicates that resistance to the phage T4 has a wide target in *E. coli* B. This is consistent with earlier studies showing that membrane modifications preventing phage adsorption represent a highly common resistance mechanism of bacteria against virulent phages and can typically be achieved by mutations in a number of genes affecting membrane structure and components (Labrie et al. 2010). Moreover, the phage caused an increase of one nonsynonymous mutation in the median mutation count of the clones, suggesting that individual mutations rather than several mutations in combination were required for phage resistance. The potential phage resistance targets discovered in this study encompass 14 genes: *acrR*, *asmA*, *fabR*, *galU*, *infB*, *lpcA*, *mscM*, *rfaQ*, ECB RS0200, ECB RS03400, ECB RS03925, ECB RS05770, ECB RS09520, and ECB RS18465 (Fig. 2G). Six among these (*acrR*, *galU*, *infB*, *rfaQ*, ECB RS03400 and ECB RS09520) were also associated with resistance to a particular antimicrobial compound and have been discussed above. Among the eight remaining genes specifically associated with phage exposure and phage resistance phenotypes, mutations in four genes (*asmA*, *fabR*, *mscM* and ECB RS18465) were particularly prevalent, with mutations in *mscM* being prevalent across antimicrobial compound environments but absent from the antimicrobial-free control environment. The product of *asmA* is involved in the assembly of outer membrane proteins and has been implicated in phage resistance (Misra and Miao 1995). The product of *fabR* is an HTH-type transcriptional repressor involved in unsaturated fatty acid biosynthesis and the physical properties of the cell membrane. Recently, both *fabR* (Yang et al. 2019) and phage resistance (Cheng et al. 2020) have more specifically been linked to L-threonine production. The gene *mscM* encodes a mechanosensitive channel protein located in the cell membrane. The channel exhibits long-term open states in response to increased membrane tension (Schumann et al. 2010), suggesting that

loss-of-function mutations may be beneficial in the presence of both phage and antimicrobial compounds if low antimicrobial concentrations increase membrane tension and open states facilitate phage adsorption. Finally, ECB RS18465 encodes a putative glucosyltransferase. Glycosyltransferases have been implicated in bacterial phage resistance through determining the glycosylation pattern of cell membrane teichoic acids required for phage adsorption (Li et al. 2015).

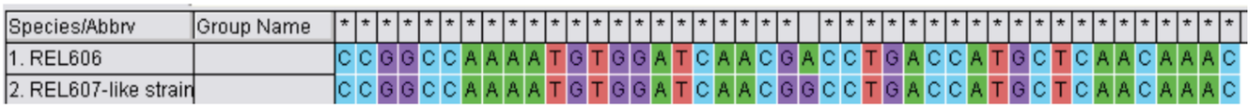

Supplementary Figure S1. Confirmation of REL607-like Ara+ reversion mutation. Related to Figure 2, Figure 3, Figure 4, and Figure 5. The figure is based on ClustalW alignment in MEGA7.

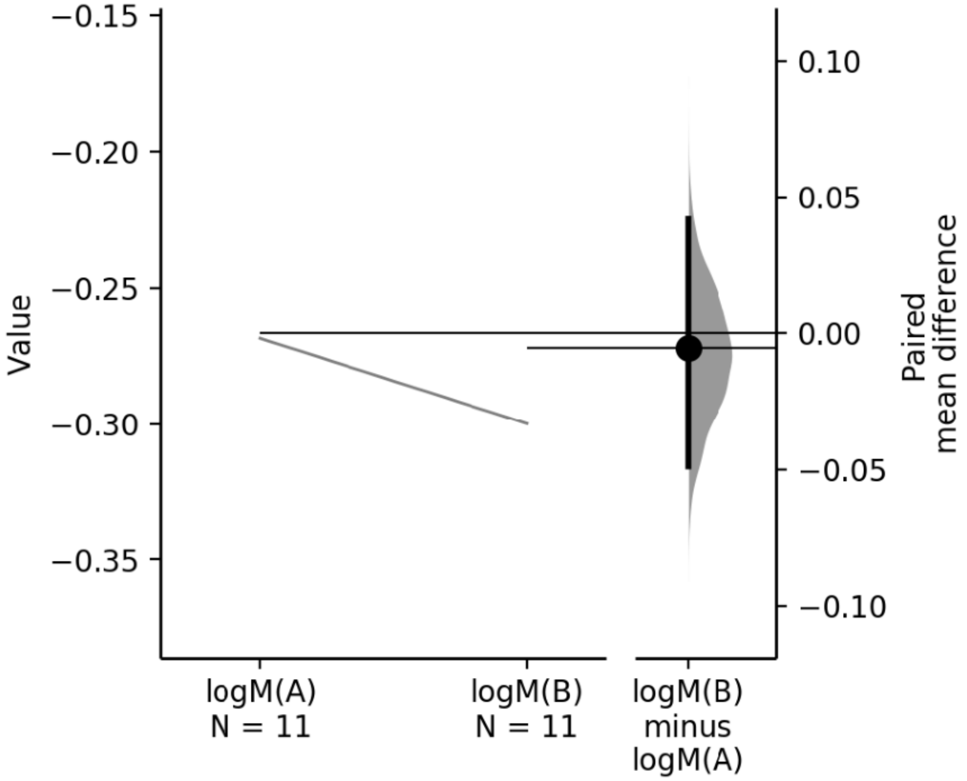

Supplementary Figure S2. Malthusian parameters for the two ancestral strains in the serial passage experiment showing lack of difference in initial fitness. Related to Figure 2, Figure 3, Figure 4, and Figure 5. The paired mean difference between log(MREL606) (A) and log(MREL607-like) (B) is shown in the above Gardner-Altman estimation plot. Both groups are plotted on the left axes as a slopegraph: each paired set of observations is connected by a line. The paired mean difference is plotted on a floating axis on the right as a bootstrap sampling distribution. The mean difference is depicted as a dot; the 95 % confidence interval is indicated by the ends of the vertical error bar.

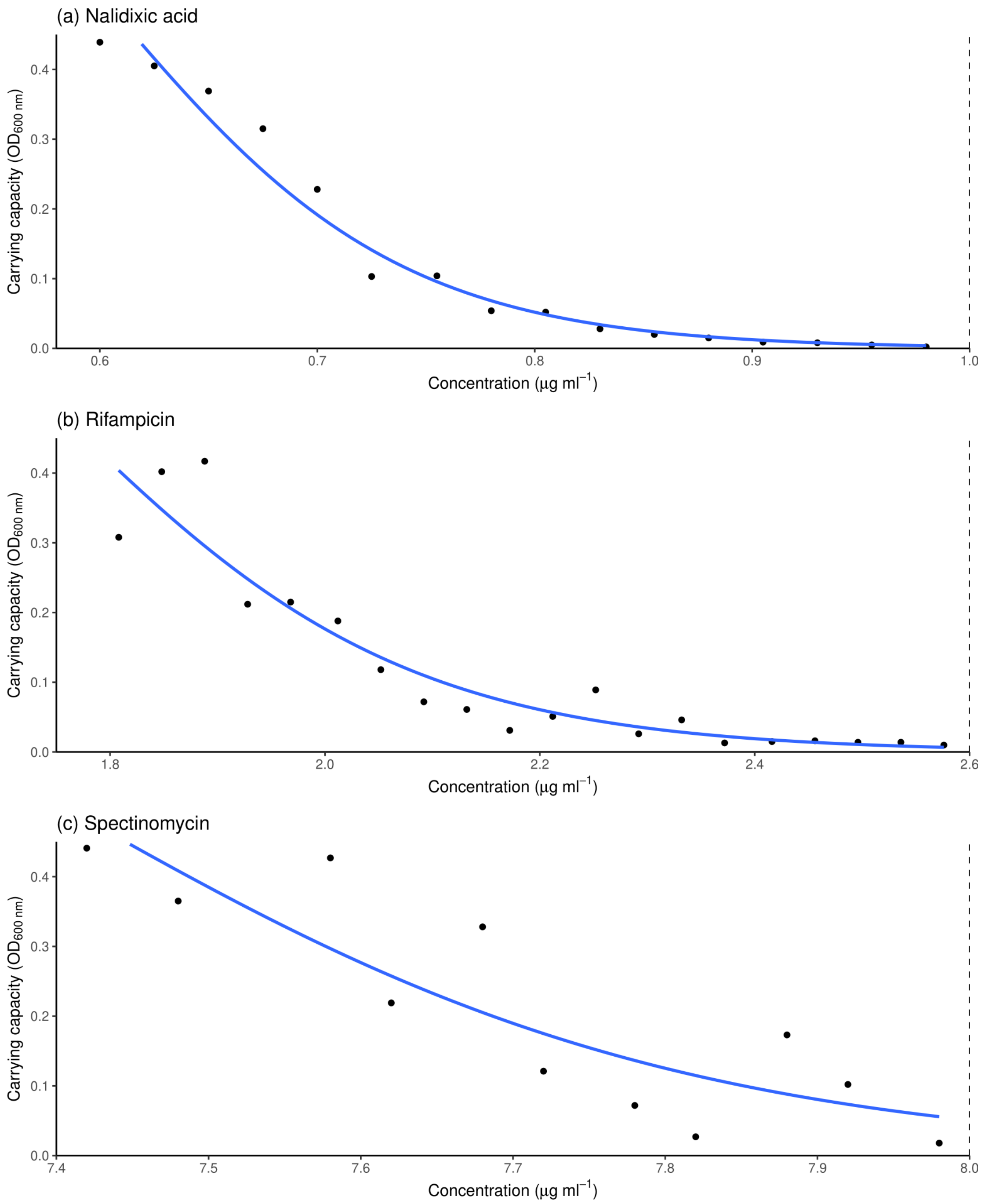

Supplementary Figure S3. Dose response curve for REL606 carrying capacity in the experimental antimicrobials. Related to Figure 2, Figure 3, Figure 4, and Figure 5. (A) nalidixic acid, (B) rifampicin, and (C) spectinomycin. The minimum inhibitory concentration (MIC) where growth is no longer observed is shown with a dashed line. The blue line is a logistic regression curve fitted to the data.

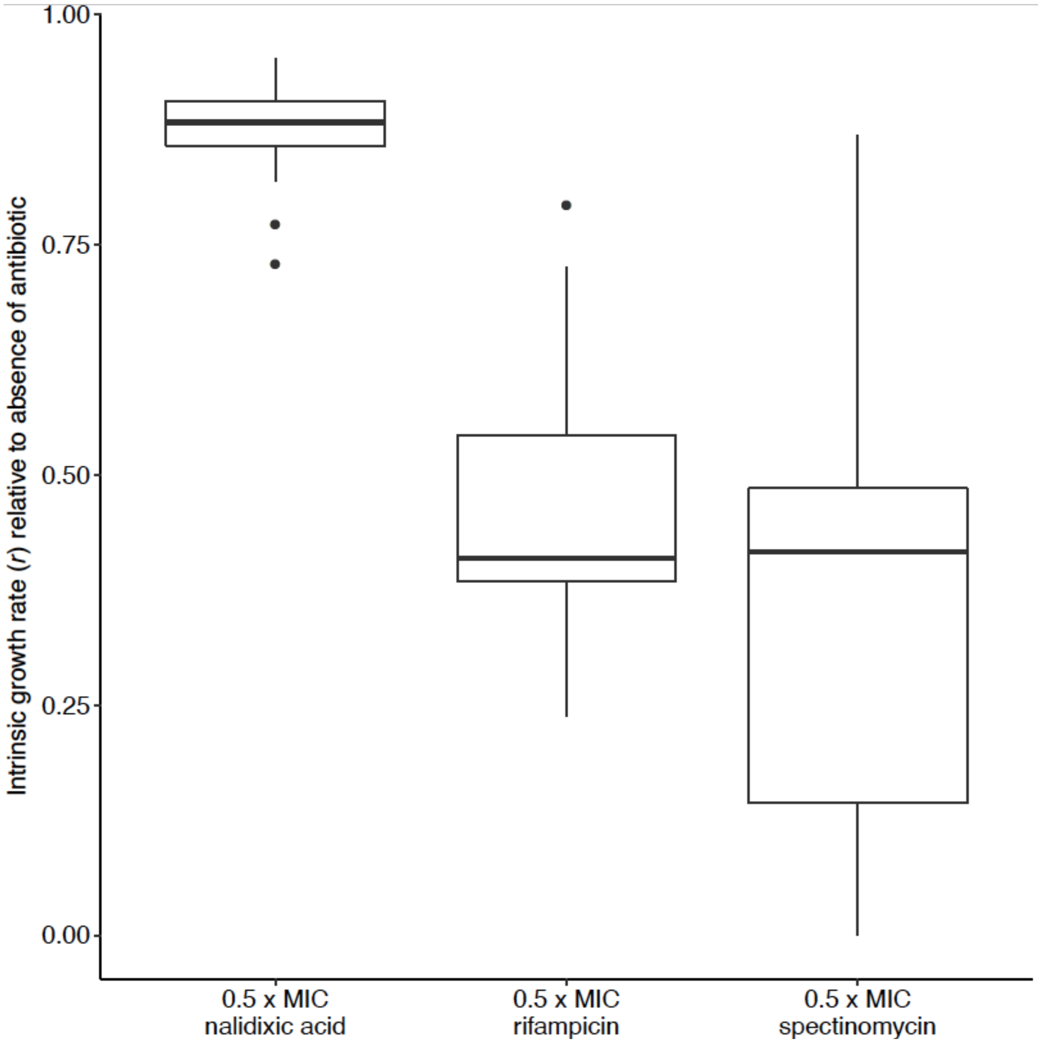

Supplementary Figure S4. Intrinsic growth rate (*r*) of ancestral REL606 in the experimental antimicrobial concentrations. The data is based on 50 technical replicate measurements for each condition from culturing for 48 h at 37 °C using the Bioscreen C well-plate reader (Labsystems, Helsinki, Finland) that measures optical density (OD) at 420–580 nm with a wideband filter at 5 min intervals. The y-axis displays intrinsic growth rate (*r*) relative to the mean value in the antibiotic-free environment. Carrying capacity (*k*) was unaffected by the experimental antibiotic concentrations.

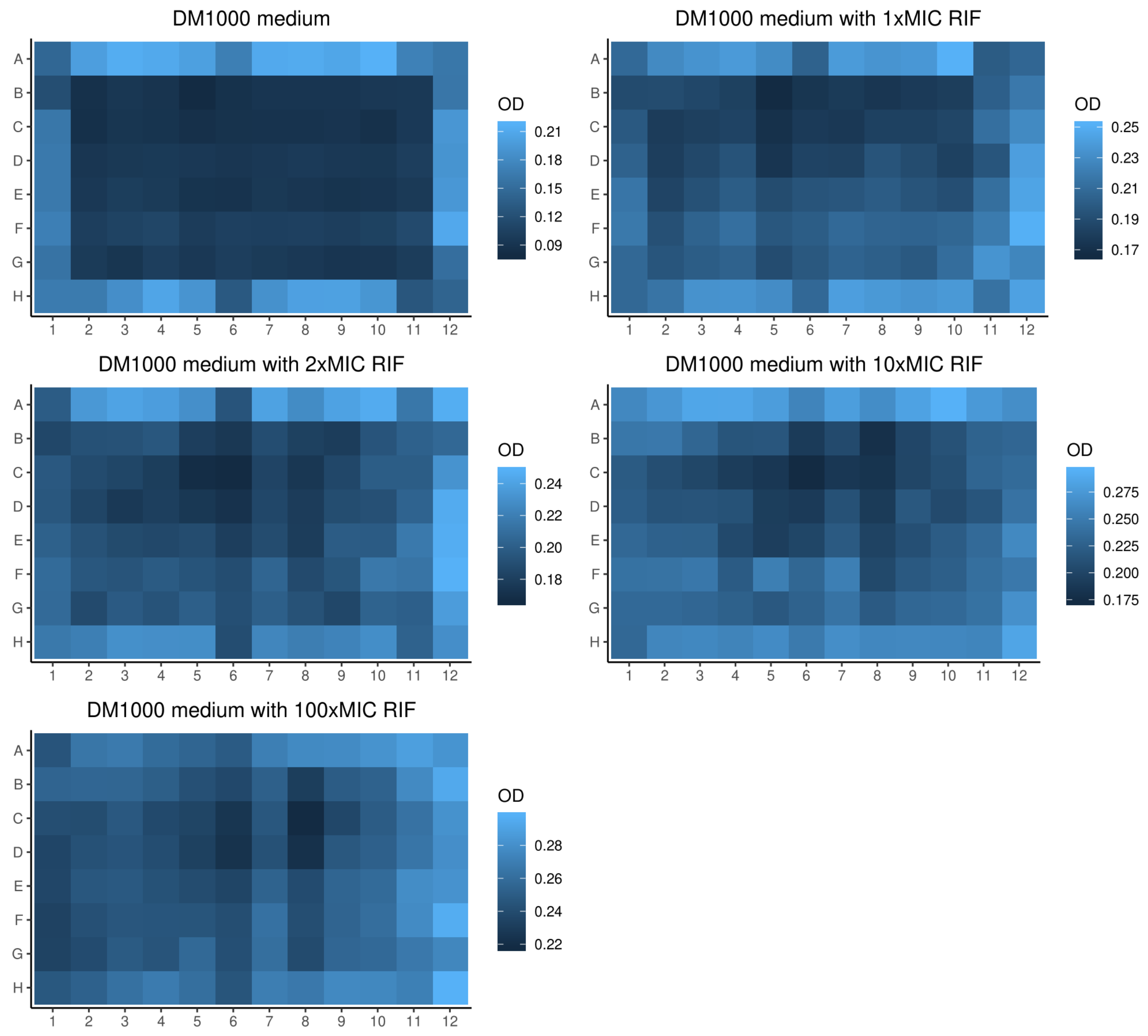

Supplementary Figure S5. Effect of well plate location on background optical density (OD) value at 600 nm obtained from the Tecan Infinite M200 device used to quantify the growth of end-point clones from serial passage experiment. Related to Figure 2, Figure 3, Figure 4, and Figure 5. The effect is shown separately for the DM1000 medium alone and with different levels of rifampicin, which affected the background OD level. The heat map displays the mean of 10 measurements per well per condition.

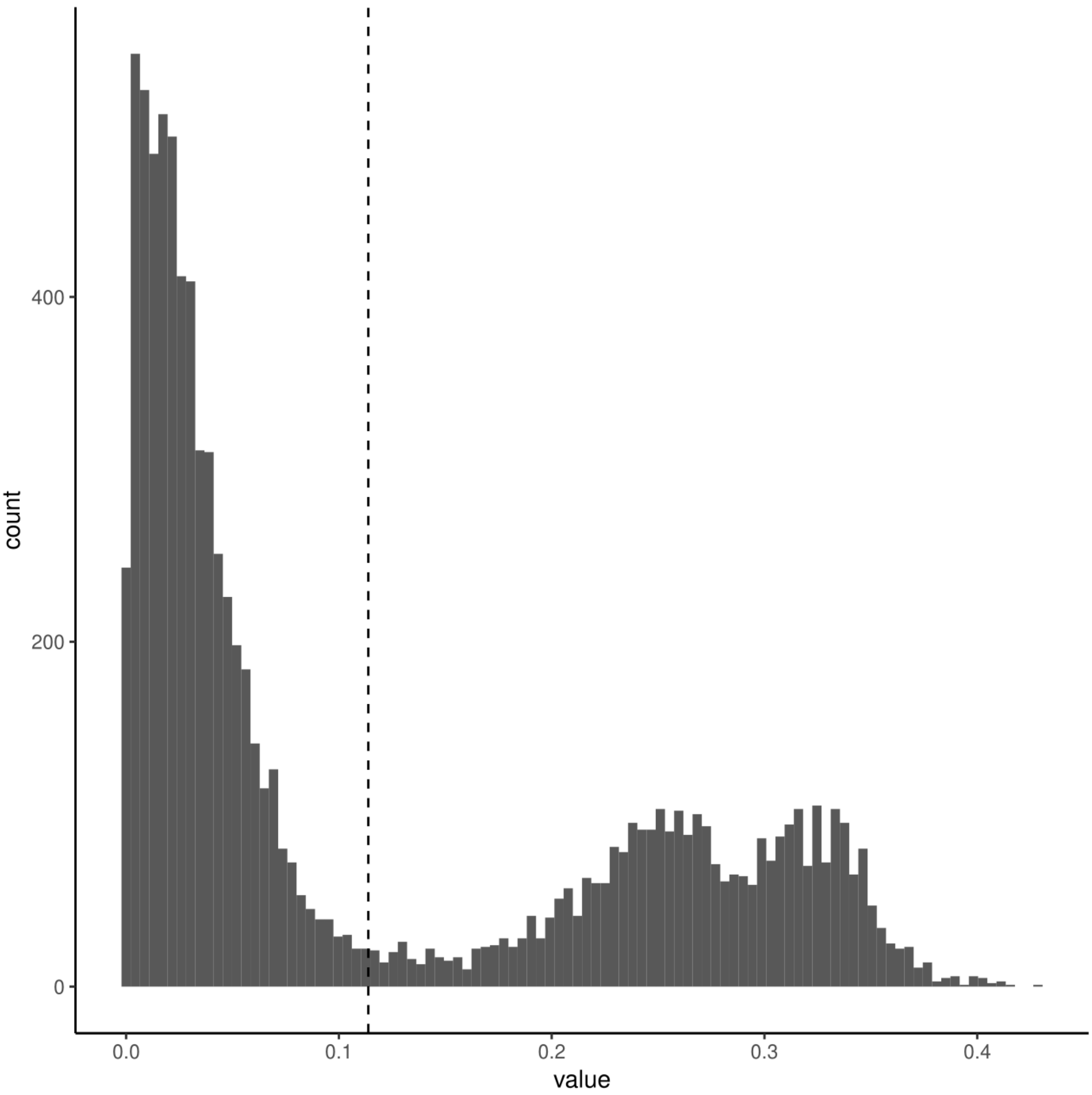

Supplementary Figure S6. Distribution of OD data for clonal lines after removing well-specific background OD values. Related to Figure 2, Figure 3, Figure 4, and Figure 5. The *x*-axis displays OD at 600 nm. The cut-off used to remove false positives (3 × standard deviation of lowest distribution mirrored on each side of zero) is indicated with a dashed line. All growth data below the cut-off value was transformed to zero growth.

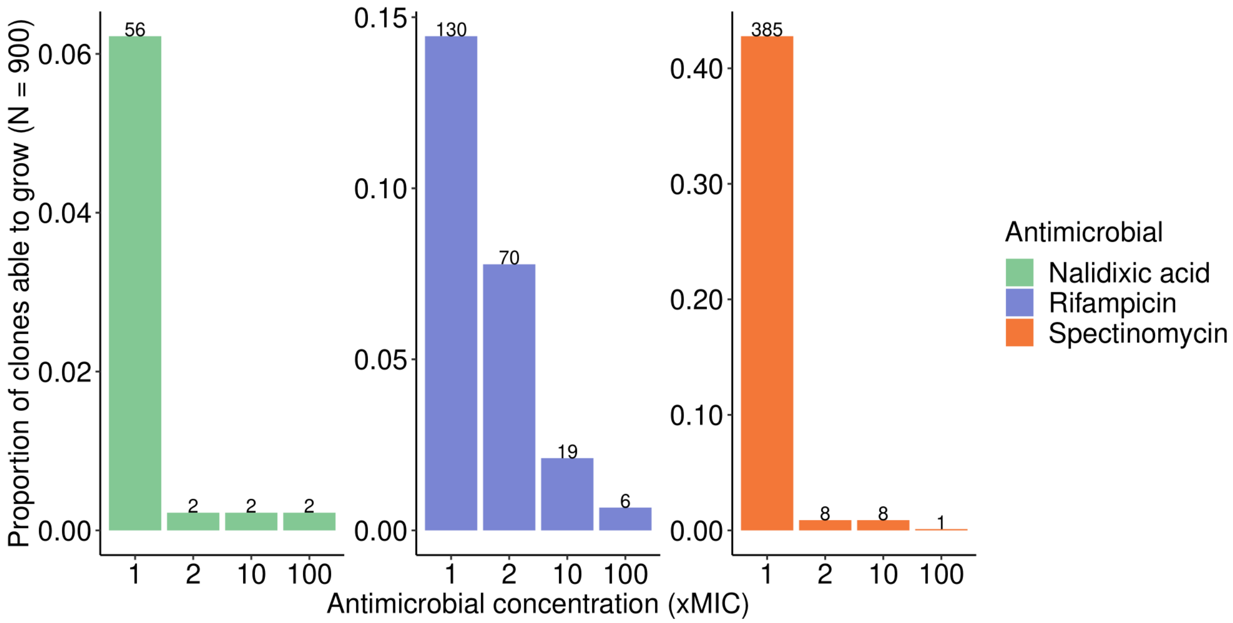

Supplementary Figure S7. Ability of clones isolated from the end-point of evolutionary experiment to grow at different concentrations of the experimental antimicrobials. Related to Figure 2, Figure 3, Figure 4, and Figure 5. The *y*-axis shows the proportion of clones and the numbers on top of bars the number of clones ($N = 900$).

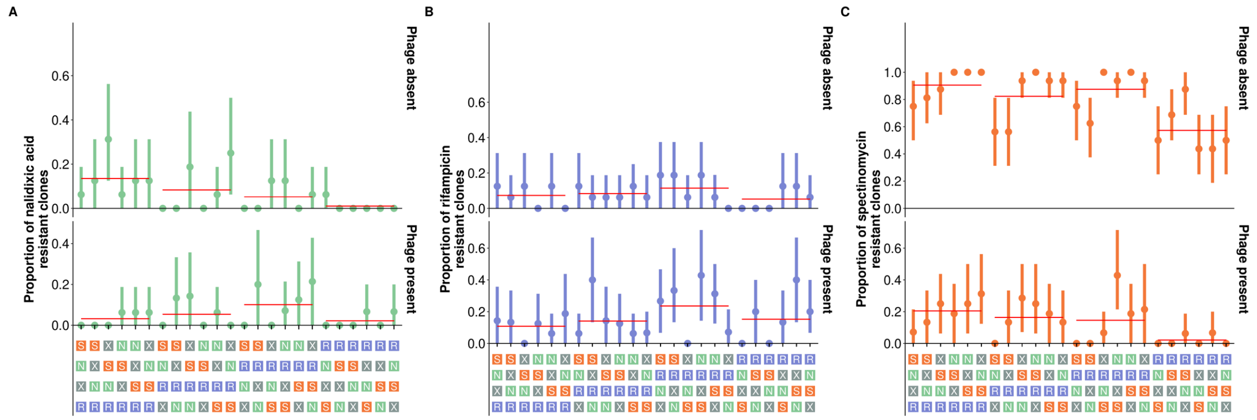

Supplementary Figure S8. Antimicrobial resistance spectra in different time-dependent regimes. Related to Figure 2, Figure 3, Figure 4, and Figure 5. (A), (B) and (C) show resistance spectra for nalidixic acid, rifampicin and spectinomycin, respectively, at the experimental end-point depending on antimicrobial sequence and presence of phage (mean resistance ± bootstrapped 95 % confidence intervals). The data is based on $N = 900$ clones isolated from populations at the experimental end point. The antimicrobial sequences have been ordered by the rifampicin exposure epoch explaining resistance to all three antimicrobials, with the mean resistance level indicated by a red line.

Supplementary Table S1. Related to Figure 2, Figure 3, Figure 4, and Figure 5. Davis-Mingioli medium with 1000 mg L^{-1} glucose (DM1000) for culturing strains in liquid medium during the competition assay, serial passage experiment, and clone measurements. Culture conditions: 24 h at 37 °C. The medium is prepared by adding dH_2O to final volume and autoclaving, followed by adding 0.5 mL each of the following stock solutions: 10 % (w/v) magnesium sulfate $MgSO_4$ (separately autoclaved stock) and 0.2 % (w/v) thiamine (vitamin B1; filter sterilized), and 5 mL of 10 % glucose (separately autoclaved stock). Sources: Levin et al. (1977); Lenski lab website (myxo.css.msu.edu/ecoli/dm25liquid.html, accessed 2018-05-04); and Barrick lab website (barricklab.org/twiki/bin/view/Lab/ProtocolsRecipesDavisMingioli, accessed 2018-06-20; barricklab.org/twiki/bin/view/Lab/ProtocolsFluctuationTests, accessed 2018-06-26).

**Component**	**0.5 L**
Potassium phosphate (dibasic) K_2HPO_4*	2.65 g
Potassium phosphate (monobasic anhydrous) KH_2PO_4	1 g
Ammonium sulfate $(NH_4)_2SO_4$	0.5 g
Sodium citrate (trisodium, dihydrate) $Na_3C_6H_5O_7 \times 2(H_2O)$	0.25 g

*If using potassium phosphate (dibasic) trihydrate ($K_2HPO_4 \times 3H_2O$), use 7 g L^{-1}.

Supplementary Table S2. Related to Figure 2, Figure 3, Figure 4, and Figure 5. Defined minimal arabinose (MA) agar for obtaining new Ara^{+} mutant from Ara^{-} parent. Culture conditions: 48 h at 37 °C. The water is split into three parts, salts, agar, and sugar, which are autoclaved separately and combined, followed by the addition of 1 mL of each of the following stock solutions: 10 % (w/v) magnesium sulfate $MgSO_4$ (separately autoclaved stock) and 0.2 (w/v) thiamine (vitamin B1; filter sterilized). Source: Lenski lab website (http://myxo.css.msu.edu/ecoli/dmagar.html, accessed 2018-05-04).

**Component**	**1 L**
Potassium phosphate (dibasic) K_2HPO_4*	5.3 g
Potassium phosphate (monobasic anhydrous) KH_2PO_4	2 g
Ammonium sulfate $(NH_4)_2SO_4$	1 g
Sodium citrate (trisodium, dihydrate) $Na_3C_6H_5O_7 \times 2(H_2O)$	0.5 g
dH_2O	1000 mL
Agar	16 g
Antifoam (5 %)	1 mL
l(+)Arabinose	4 g

*If using potassium phosphate (dibasic) trihydrate ($K_2HPO_4 \times 3H_2O$), use 7 g L^{-1}.

Supplementary Table S3. Related to Figure 2, Figure 3, Figure 4, and Figure 5. Recipe for tetrazolium and arabinose (TA) agar plates for distinguishing between Ara^- (red/purple) and Ara^+ (white/pink) strains. Culture conditions: 24 h at 37 ºC. The medium is prepared by splitting the water, autoclaving the arabinose separately, combining the two parts after autoclaving, adding TTC from a sterile stock solution, and mixing. Sources: Carlton and Brown (1981) and Lenski lab website (http://myxo.css.msu.edu/ecoli/taagar.html, accessed 2018-05-04).

**Component**	**1 L**	
Tryptone	10 g	
Yeast extract	1 g	
Sodium chloride NaCl	5 g	
Agar	16 g	
Antifoam (5 %)	1 mL	
dH_2O	1000 mL	
l(+)Arabinose	10 g	
TTC (5 %) $C_{19}H_{15}ClN_4$	1 mL	TTC is the tetrazolium indicator dye

Supplementary Table S4. Related to Figure 2, Figure 3, Figure 4, and Figure 5. ANOVA table for generalized least squares model on temporal nalidixic acid resistance dynamics at population level.

**Model term**	***Df***	***F***	***P***
Antimicrobial regime (control / time-invariant single-agent / time-invariant combination / time-dependent)	3	29.3	< 0.001
Phage (present/absent)	1	32.4	< 0.001
Antimicrobial regime × phage	3	0.39	0.76
Residuals	3348		

Supplementary Table S5. Related to Figure 2, Figure 3, Figure 4, and Figure 5. ANOVA table for binomial generalized linear (*i.e.* logistic regression) model on nalidixic acid resistance levels in end-point clones.

**Model term**	***Df***	**Deviance**	**Residual *df***	***P* (χ^2)**
Antimicrobial regime (control / time-invariant single-agent / time-invariant combination / time-dependent)	3	11.9	835	0.008
Phage (present/absent)	1	0.041	834	0.84
Antimicrobial regime × phage	3	6.80	831	0.079

Supplementary Table S6. Related to Figure 2, Figure 3, Figure 4, and Figure 5. ANOVA table for generalized least squares model on temporal rifampicin resistance dynamics at population level.

**Model term**	***Df***	***F***	***P***
Antimicrobial regime (control / time-invariant single-agent / time-invariant combination / time-dependent)	3	29.3	< 0.001
Phage (present/absent)	1	5.49	0.019
Antimicrobial regime × phage	3	1.58	0.19
Residuals	3340		

Supplementary Table S7. Related to Figure 2, Figure 3, Figure 4, and Figure 5. ANOVA table for binomial generalized linear (*i.e.* logistic regression) model on rifampicin resistance levels in end-point clones.

**Model term**	***Df***	**Deviance**	**Residual *df***	***P* (χ^2)**
Antimicrobial regime (control / time-invariant single-agent / time-invariant combination / time-dependent)	3	114.1	833	< 0.001
Phage (present/absent)	1	13.5	832	< 0.001
Antimicrobial regime × phage	3	0.61	829	0.89

Supplementary Table S8. Related to Figure 2, Figure 3, Figure 4, and Figure 5. ANOVA table for binomial generalized linear (*i.e.* logistic regression) model on spectinomycin resistance levels in end-point clones.

**Model term**	***Df***	**Deviance**	**Residual *df***	***P* (χ^2)**
Antimicrobial regime (control / time-invariant single-agent / time-invariant combination / time-dependent)	3	35.6	836	< 0.001
Phage (present/absent)	1	327.3	835	< 0.001
Antimicrobial regime × phage	3	32.1	832	< 0.001

Supplementary Table S9. Related to Figure 2, Figure 3, Figure 4, and Figure 5. ANOVA table for binomial generalized linear (*i.e.* logistic regression) model on phage resistance levels in end-point clones.

**Model term**	***Df***	**Deviance**	**Residual *df***	***P* (χ^2)**
Antimicrobial regime (control / time-invariant single-agent / time-invariant combination / time-dependent)	3	17.9	440	< 0.001

Supplementary Table S10. Related to Figure 2, Figure 3, Figure 4, and Figure 5. ANOVA table for binomial generalized linear (*i.e.* logistic regression) model on spectinomycin resistance levels in end-point clones comparing control and time-invariant rifampicin environments.

**Model term**	***Df***	**Deviance**	**Residual *df***	***P* (χ^2)**
Antimicrobial regime (control / time-invariant rifampicin environment)	1	4.24	59	0.039
Phage (present/absent)	1	13.3	58	<0.001
Antimicrobial regime × phage	1	9.97	57	0.002

Supplementary Table S11. Related to Figure 2, Figure 3, Figure 4, and Figure 5. ANOVA table for binomial generalized linear (*i.e.* logistic regression) model on rifampicin resistance levels in end-point clones as a function of rifampicin exposure epoch and phage exposure.

**Model term**	***Df***	**Deviance**	**Residual *df***	***P* (χ^2)**
Rifampicin exposure epoch	3	6.94	746	0.074
Phage (present/absent)	1	11.2	745	$<$ 0.001
Rifampicin exposure epoch × phage	3	1.24	742	0.74

Supplementary Table S12. Related to Figure 2, Figure 3, Figure 4, and Figure 5. ANOVA table for binomial generalized linear (*i.e.* logistic regression) model on nalidixic acid resistance levels in end-point clones as a function of nalidixic acid and rifampicin exposure epochs in the absence of phage exposure.

**Model term**	***Df***	**Deviance**	**Residual *df***	***P* (χ^2)**
Nalidixic acid exposure epoch	3	2.46	380	0.48
Rifampicin exposure epoch	3	13.9	377	0.003
Nalidixic acid exposure epoch × rifampicin exposure epoch	5	4.93	372	0.42

Supplementary Table S13. Related to Figure 2, Figure 3, Figure 4, and Figure 5. ANOVA table for binomial generalized linear (*i.e.* logistic regression) model on nalidixic acid resistance levels in end-point clones as a function of nalidixic acid and rifampicin exposure epochs in the presence of phage exposure.

**Model term**	***Df***	**Deviance**	**Residual *df***	***P* (χ^2)**
Nalidixic acid exposure epoch	3	12.2	362	0.007
Rifampicin exposure epoch	3	4.37	359	0.22
Nalidixic acid exposure epoch × rifampicin exposure epoch	5	10.2	354	0.069

Supplementary Table S14. Related to Figure 2, Figure 3, Figure 4, and Figure 5. ANOVA table for binomial generalized linear (*i.e.* logistic regression) model on spectinomycin resistance levels in end-point clones as a function of spectinomycin and rifampicin exposure epochs and phage exposure.

Model term	*Df*	Deviance	Residual *df*	*P* (χ^2)
Spectinomycin exposure epoch	3	11.0	746	0.012
Rifampicin exposure epoch	3	47.5	743	< 0.001
Phage (presence/absence)	1	416.0	742	< 0.001
Spectinomycin exposure epoch × rifampicin exposure epoch	5	10.2	737	0.071
Spectinomycin exposure epoch × phage	3	1.74	734	0.63
Rifampicin exposure epoch × phage	3	1.41	731	0.70
Spectinomycin exposure epoch × rifampicin exposure epoch × phage	5	5.96	726	0.31

References

Anderson, P. 1969. Sensitivity and resistance to spectinomycin in Escherichia coli. J Bacteriol 100:939-947.

Andersson, D. I., H. Nicoloff, and K. Hjort. 2019. Mechanisms and clinical relevance of bacterial heteroresistance. Nat Rev Microbiol 17:479-496.

Carlton, B. C. and B. J. Brown. 1981. Gene mutation. Pp. 222-242 in P. Gerhardt (ed.), Manual of methods for general bacteriology. American Society for Microbiology, Washington, D.C. .

Cheng, L., J. Wang, X. Zhao, H. Yin, H. Fang, C. Lin, S. Zhang, Z. Shen, and C. Zhao. 2020. An antiphage *Escherichia coli* mutant for higher production of L-threonine obtained by atmospheric and room temperature plasma mutagenesis. Biotechnol Prog:e3058.

Cingolani, P., A. Platts, L. Wang le, M. Coon, T. Nguyen, L. Wang, S. J. Land, X. Lu, and D. M. Ruden. 2012. A program for annotating and predicting the effects of single nucleotide polymorphisms, SnpEff: SNPs in the genome of *Drosophila melanogaster* strain w1118; iso-2; iso-3. Fly (Austin) 6:80-92.

Correia, S., J. D. Nunes-Miranda, L. Pinto, H. M. Santos, M. de Toro, Y. Saenz, C. Torres, J. L. Capelo, P. Poeta, and G. Igrejas. 2014. Complete proteome of a quinolone-resistant *Salmonella* Typhimurium Phage Type DT104B Clinical Strain. Int J Mol Sci 15:14191-14219.

Culp, E. and G. D. Wright. 2017. Bacterial proteases, untapped antimicrobial drug targets. J Antibiot 70:366-377.

Girgis, H. S., A. K. Hottes, and S. Tavazoie. 2009. Genetic architecture of intrinsic antibiotic susceptibility. PLOS ONE 4:e5629.

Gliniewicz, K., M. Wildung, L. H. Orfe, G. D. Wiens, K. D. Cain, K. K. Lahmers, K. R. Snekvik, and D. R. Call. 2015. Potential mechanisms of attenuation for rifampicin-passaged strains of *Flavobacterium psychrophilum*. BMC Microbiol 15:179.

Grove, A. 2013. MarR family transcription factors. Curr Biol 23:R142-R143.

Harmand, N., R. Gallet, G. Martin, and T. Lenormand. 2018. Evolution of bacteria specialization along an antibiotic dose gradient. Evol Lett 2:221-232.

Ho, J., T. Tumkaya, S. Aryal, H. Choi, and A. Claridge-Chang. 2019. Moving beyond *P* values: data analysis with estimation graphics. Nat Methods 16:565-566.

Huseby, D. L., G. Brandis, L. P. Alzrigat, and D. Hughes. 2020. Antibiotic resistance by high-level intrinsic suppression of a frameshift mutation in an essential gene. P Natl Acad Sci USA 117:3185-3191.

Kuhn, M. Contributions from Jed Wing, S. W., Andre Williams, Chris Keefer, Allan Engelhardt, Tony Cooper, Zachary Mayer, Brenton Kenkel, the R Core Team, Michael Benesty, Reynald Lescarbeau, Andrew Ziem, Luca Scrucca, Yuan Tang and Can Candan. 2016. caret: Classification and regression training. R package version 6.0-71. https://CRAN.R-project.org/package=caret.

Kumar, S., G. Stecher, and K. Tamura. 2016. MEGA7: Molecular Evolutionary Genetics Analysis Version 7.0 for Bigger Datasets. Mol Biol Evol 33:1870-1874.

Labrie, S. J., J. E. Samson, and S. Moineau. 2010. Bacteriophage resistance mechanisms. Nat Rev Microbiol 8:317-327.

Langmead, B. and S. L. Salzberg. 2012. Fast gapped-read alignment with Bowtie 2. Nat Methods 9:357-359.

Lazar, V., G. P. Singh, R. Spohn, I. Nagy, B. Horvath, M. Hrtyan, R. Busa-Fekete, B. Bogos, O. Mehi, B. Csorgo, G. Posfai, G. Fekete, B. Szappanos, B. Kegl, B. Papp, and C. Pal. 2013. Bacterial evolution of antibiotic hypersensitivity. Mol Syst Biol 9:700.

Le, S., X. Y. Yao, S. G. Lu, Y. L. Tan, X. C. Rao, M. Li, X. L. Jin, J. Wang, Y. Zhao, N. C. Wu, R. Lux, X. S. He, W. Y. Shi, and F. Q. Hu. 2014. Chromosomal DNA deletion confers phage resistance to *Pseudomonas aeruginosa*. Sci Rep 4:4738.

Levin, B. R., F. M. Stewart, and L. Chao. 1977. Resource-limited growth, competition, and predation: A model and experimental studies with bacteria and bacteriophage. Am Nat 111:3-24.

Li, H., B. Handsaker, A. Wysoker, T. Fennell, J. Ruan, N. Homer, G. Marth, G. Abecasis, R. Durbin, and S. Genome Project Data Processing. 2009. The Sequence Alignment/Map format and SAMtools. Bioinformatics 25:2078-2079.

Li, X. H., D. Gerlach, X. Du, J. Larsen, M. Stegger, P. Kuhner, A. Peschel, G. Q. Xia, and V. Winstel. 2015. An accessory wall teichoic acid glycosyltransferase protects *Staphylococcus aureus* from the lytic activity of Podoviridae. Sci Rep 5:17219.

Mahto, A. 2019. splitstackshape: Stack and reshape datasets after splitting concatenated values. R package version 1.4.8.

Mariam, D. H., Y. Mengistu, S. E. Hoffner, and D. I. Andersson. 2004. Effect of *rpoB* mutations conferring rifampin resistance on fitness of *Mycobacterium tuberculosis*. Antimicrob Agents Chemother 48:1289-1294.

McKenna, A., M. Hanna, E. Banks, A. Sivachenko, K. Cibulskis, A. Kernytsky, K. Garimella, D. Altshuler, S. Gabriel, M. Daly, and M. A. DePristo. 2010. The Genome Analysis Toolkit: a MapReduce framework for analyzing next-generation DNA sequencing data. Genome Res 20:1297-1303.

Meyer, J. R., I. Gudelj, and R. Beardmore. 2015. Biophysical mechanisms that maintain biodiversity through trade-offs. Nat Commun 6:6278.

Misra, R. and Y. Miao. 1995. Molecular analysis of *asmA*, a locus identified as the suppressor of Ompf assembly mutants of *Escherichia coli* K-12. Mol Microbiol 16:779-788.

Nicoloff, H., K. Hjort, B. R. Levin, and D. I. Andersson. 2019. The high prevalence of antibiotic heteroresistance in pathogenic bacteria is mainly caused by gene amplification. Nat Microbiol 4:504-514.

Oz, T., A. Guvenek, S. Yildiz, E. Karaboga, Y. T. Tamer, N. Mumcuyan, V. B. Ozan, G. H. Senturk, M. Cokol, P. Yeh, and E. Toprak. 2014. Strength of selection pressure is an important parameter contributing to the complexity of antibiotic resistance evolution. Mol Biol Evol 31:2387-2401.

Paterson, G. K., D. B. Cone, S. E. Peters, and D. J. Maskell. 2009. The enzyme phosphoglucomutase (Pgm) is required by *Salmonella enterica* serovar Typhimurium for O-antigen production, resistance to antimicrobial peptides and in vivo fitness. Microbiol-Sgm 155:3403-3410.

Poole, K. 2012. Bacterial stress responses as determinants of antimicrobial resistance. J Antimicrob Chemoth 67:2069-2089.

Rodriguez-Verdugo, A., B. S. Gaut, and O. Tenaillon. 2013. Evolution of *Escherichia coli* rifampicin resistance in an antibiotic-free environment during thermal stress. BMC Evol Biol 13:50.

Schneiders, T., S. G. B. Amyes, and S. B. Levy. 2003. Role of AcrR and RamA in fluoroquinolone resistance in clinical *Klebsiella pneumoniae* isolates from Singapore. Antimicrob Agents Chemother 47:2831-2837.

Schumann, U., M. D. Edwards, T. Rasmussen, W. Bartlett, P. van West, and I. R. Booth. 2010. YbdG in *Escherichia coli* is a threshold-setting mechanosensitive channel with MscM activity. P Natl Acad Sci USA 107:12664-12669.

Staden, R., K. F. Beal, and J. K. Bonfield. 2000. The Staden package, 1998. Methods Mol Biol 132:115-130.

Strugeon, E., V. Tilloy, M. C. Ploy, and S. Da Re. 2016. The stringent response promotes antibiotic resistance dissemination by regulating integron integrase expression in biofilms. mBio 7:e00868-16.

Yang, J., Y. Fang, J. Wang, C. Wang, L. Zhao, and X. Wang. 2019. Deletion of regulator-encoding genes *fadR*, *fabR* and *iclR* to increase L-threonine production in *Escherichia coli*. Appl Microbiol Biotechnol 103:4549-4564.

Zhang, Y., E. Zbornikova, D. Rejman, and K. Gerdes. 2018. Novel (p)ppGpp binding and metabolizing proteins of *Escherichia coli*. mBio 9:e02188-17.

Zorzet, A., M. Y. Pavlov, A. I. Nilsson, M. Ehrenberg, and D. I. Andersson. 2010. Error-prone initiation factor 2 mutations reduce the fitness cost of antibiotic resistance. Mol Microbiol 75:1299-1313.
